# Endogenous single-strand DNA breaks at RNA polymerase II promoters in *Saccharomyces cerevisiae*

**DOI:** 10.1093/nar/gky743

**Published:** 2018-08-24

**Authors:** Éva Hegedüs, Endre Kókai, Péter Nánási, László Imre, László Halász, Rozenn Jossé, Zsuzsa Antunovics, Martin R Webb, Aziz El Hage, Yves Pommier, Lóránt Székvölgyi, Viktor Dombrádi, Gábor Szabó

**Affiliations:** 1 Department of Biophysics and Cell Biology, Faculty of Medicine, University of Debrecen, Debrecen, Hungary; 2 Department of Medical Chemistry, Faculty of Medicine, University of Debrecen, Debrecen, Hungary; 3 MTA-DE Momentum Genome Architecture and Recombination Research Group, Department of Biochemistry and Molecular Biology, Faculty of Medicine, University of Debrecen, Debrecen, Hungary; 4 Developmental Therapeutics Branch and Laboratory of Molecular Pharmacology, Center for Cancer Research, National Cancer Institute (CCR-NCI), NIH, Bethesda, MD, USA; 5 Department of Genetics and Applied Microbiology, Faculty of Science and Technology, University of Debrecen, Debrecen, Hungary; 6 The Francis Crick Institute, London NW1 1AT, UK; 7 Wellcome Trust Centre for Cell Biology, University of Edinburgh, Edinburgh, UK

## Abstract

Molecular combing and gel electrophoretic studies revealed endogenous nicks with free 3′OH ends at ∼100 kb intervals in the genomic DNA (gDNA) of unperturbed and G1-synchronized *Saccharomyces cerevisiae* cells. Analysis of the distribution of endogenous nicks by Nick ChIP-chip indicated that these breaks accumulated at active RNA polymerase II (RNAP II) promoters, reminiscent of the promoter-proximal transient DNA breaks of higher eukaryotes. Similar periodicity of endogenous nicks was found within the ribosomal rDNA cluster, involving every ∼10th of the tandemly repeated 9.1 kb units of identical sequence. Nicks were mapped by Southern blotting to a few narrow regions within the affected units. Three of them were overlapping the RNAP II promoters, while the ARS-containing IGS2 region was spared of nicks. By using a highly sensitive reverse-Southwestern blot method to map free DNA ends with 3′OH, nicks were shown to be distinct from other known rDNA breaks and linked to the regulation of rDNA silencing. Nicks in rDNA and the rest of the genome were typically found at the ends of combed DNA molecules, occasionally together with R-loops, comprising a major pool of vulnerable sites that are connected with transcriptional regulation.

## INTRODUCTION

Recent observations in several mammalian experimental models suggest that transcriptional activation of RNA polymerase II (RNAP II) dependent genes frequently involves formation of DNA strand breaks, supposedly elicited by topoisomerase 2β (Top 2β) (1–7). Indeed, this enzyme has been mapped to the 5′ end of transcriptionally active genes in several experimental systems (8–10). These breakages are generally interpreted in the context of the requirement for topological relaxation during transcription, although elongation by RNAP rather than initiation is readily explained in terms of the twin supercoil domain model (11–13), while strand opening at initiation is facilitated by negative supercoiling (14,15). Transient discontinuities have been detected at the promoters of several genes by 3′OH end-labeling; these are generally perceived as double strand (ds) breaks even though the two subunits of Top2 are known to work independently of each other and the DNA discontinuities generated by this enzyme are partly single-strand (ss) breaks/nicks (9,16). Furthermore, DNA is sensitive to mechanical damage at ss breaks (data in (17)), so ds breaks may be indirectly generated at the site of Top2 activity. Mechanical breakages may include those that arise due to an abrupt release of torsional stress upon deproteinization. On the other hand, non-random ss breaks may also arise when topoisomerases, and perhaps some other nucleases present in a cell lysate, might cleave one strand at accessible sites so that the relaxed DNA becomes resistant to further cleavages (similarly to the observations in (18)), that would result in a single nick in each supercoiled loop. In addition, many restriction enzymes generate ss incisions at imperfect recognition motifs (19,20), that are easily mistaken for endogenous breaks. Furthermore, the 3′ end-labeling procedures may also be misleading since the 3′end of the RNA moiety in R-loops (three-stranded structures composed of an RNA:DNA hybrid and a displaced ss DNA strand (21,22)) could also serve as starting points for either terminal transferase (23) or DNA polymerase activity (24) that may be mistakenly interpreted as nicks. In the light of these experimental challenges, the findings on endogenous breaks are often regarded as controversial and clarification of the questions concerning their origin and function calls for alternative, independent approaches. To overcome these caveats, one possibility would be to have a global view on all of the endogenous nicks in the genome rather than focusing on particular loci, in better known model organisms, using novel methods.

It has been reported in our earlier published studies that conventional procedures involving extensive proteolytic digestion of lysed cells followed by phenol–chloroform extraction or purification based on silica-adsorption, yield ∼50 kb ds DNA fragments (25,26). Remarkably, this average fragment size overlaps that of the DNA loops (27) and coincides with the estimated size of the functional (transcriptional, replicative) units of chromatin (28–32). According to the common view, such loop-size ds fragmentation could be a direct consequence of random mechanical breakage, since it can be avoided when the cells are embedded into agarose plugs before lysis. Importantly however, a similar fragmentation was observed even when the DNA in agarose plugs was treated with S1 nuclease (33), an enzyme that preferentially cleaves at nicks and ss DNA regions (34). Moreover, loop-size fragmentation occurs upon rapid alkaline lysis of cells (26,35), upon urea/heat-denaturation of intact chromatin embedded in agarose (36,37), and also when DNA is isolated from fixed cells (38). Using *in situ* nick-translation and single-cell gel electophoresis we have previously shown that preformed ss discontinuities, i.e. nicks are scattered over eukaryotic chromatin, including that of *Saccharomyces cerevisiae*, delimiting loop-size domains (37). All these observations could be collectively interpreted in terms of persistent, endogenous ss breaks that may yield ds breaks depending on the experimental conditions. We hypothesize that the promoter-proximal, transcriptional activity-related DNA breaks that have been reported in mammalian cells (reviewed in (39)) represent a major subpopulation of the endogenous nicks detected by us at loop-size intervals in various eukaryotic systems (26,33,36–38).

Based on this hypothesis, we exploited the advantages offered by assessing endogenous DNA breaks in a global manner over the genomic DNA (gDNA) of *S. cerevisiae*. To investigate the possible sequence-related and sequence-unrelated determinants of the breaks, the rDNA cluster containing naturally amplified units of identical sequence was also included in our study. The ∼1–2 Mb rDNA cluster is located at the *RDN1* locus in the right arm of chromosome XII (chr XII) in budding yeast and consists of a tandem array of 100–200 repeated transcription units. The rDNA organizes the nucleolus wherein transcription of ribosomal RNA (rRNA) and the early steps of ribosome biogenesis take place (40,41). A perinucleolar protein network including the replication fork barrier (RFB)-associated protein Fob1 and the topoisomerase 1 enzyme (Top1) tethers the rDNA repeats to the nuclear membrane, thus separating the locus from the bulk DNA inside the nucleus, and also ensures repeat stability (42). Top1mediates ss nicking and re-ligation in a process involving stabilized Top1 covalent cleavage complexes (Top1cc) formed *via* the 3′OH of the nicked DNA (17).

The 9.1 kb rDNA units (see Figure 4A) harbour the genes for 5.8S, 25S and 18S rRNAs which are transcribed by RNAP I as a single precursor (i.e. 35S pre-rRNA). Each unit contains also a 5S rRNA gene transcribed by RNAP III from the opposite strand. A replication origin (rARS element) is positioned outside the transcribed regions within IGS2. Although DNA replication begins bidirectionally, the rightward-moving fork is arrested at the RFB within IGS1, while the other fork proceeds through about five repeats until it terminates at a stalled rightward-moving fork of the closest replication unit. These RFBs prevent the replisome from head-on collison with the RNAP I transcription apparatus, so that rRNA transcription can proceed even during S-phase (43). About half of the units are transcriptionally active at the same time (44–46) and are transcribed at high efficiency by ∼50 RNAP I complexes per 35S pre-rRNA (47). About 20% of all available rARSs are used as replication origins during a single S phase (48). There are also a few genes that are transcribed by RNAP II within the rDNA units. Accordingly, RNAP II can be detected by chromatin immunoprecipitation (ChIP) in the corresponding promoter regions (49). A preferential association of DNA breaks with the rARS regions or with the regulatory regions of any of the transcribed genes would indicate that the mechanism that generates the DNA breaks is associated with rDNA replication or transcription, respectively.

Here, we made use of enzymatic labeling of endogenous free 3′OH ends in conjunction with molecular combing, microarray analyses, and a novel reverse Southwestern (rSW) blotting procedure in order to characterize the endogenous nicks. The nicks were mapped genome wide, including the repetitive rDNA locus. The specific localization of the nicks together with the comparison of mutant yeast strains suggest that their biological function is associated with transcription. Importantly also, the picture that emerges from our observations implicates these DNA breaks in genomic instability.

## MATERIALS AND METHODS

For *cultivation and* s*ynchronization of S. cerevisiae and S. pombe and description of the common gel electrophoretic techniques* see Supplementary Materials and Methods. The budding yeast strains used in the present study are listed in Table 1.

**Table 1.** List of *S. cerevisiae* strains used in this study

Strain	Genomic background	Genotype	Source
WDHY199	W303	Mat a, leu2-3,112, trp1-283, ura3-52, his7-2, lys1-1	Wolf-Dietrich Heyer lab
BY4741	WT	Mat a, his3Δ1, leu2Δ0, met15Δ0, ura3Δ0	EUROFAN
*top1Δ*	BY4741	Mat a, his3Δ1, leu2Δ0, met15Δ0, ura3Δ0, top1Δ::KanMX	EUROFAN
*bar1Δ*	BY4741	Mat a; his3D1; leu2D0; met15D0; ura3D0; YIL015wΔ::KanMX4	EUROFAN
*fob1Δ*	BY4741	Mat a, his3Δ1, leu2Δ0, met15Δ0, ura3Δ0, fob1:: KanMX4	EUROFAN
*sir2 Δ*	BY4741	Mat a, his3Δ1, leu2Δ0, met15Δ0, ura3Δ0, sir2:: KanMX4	EUROFAN

### Preparation of agarose plugs containing yeast chromosomes

Preparation of *S. cerevisiae* agarose-plugs was carried out as previously described (33). Briefly, *S. cerevisiae* cells were harvested and washed twice in 50 mM EDTA (pH 8.0), then resuspended in digestion solution (0.9 M sorbitol, 0.125 M EDTA,100 mM dithiothreitol (DTT)) containing 2 mg/ml lyticase enzyme (Sigma-Aldrich). Samples were mixed with an equal volume of 1.5% low melting point (LMP) agarose (Sigma-Aldrich) dissolved in 0.9 M sorbitol/0.125 M EDTA. Aliquots were allowed to harden in sample molds at 4°C for 5 min, and then placed into 0.9 M sorbitol/0.125 M EDTA at 37°C for 6 h. Each plug contained ∼3 × 10^8^ cells. The plugs containing yeast spheroplasts were digested with 0.5 mg/ml Proteinase K (Thermo Fisher Scientific) in lysing solution (0.5 M EDTA, 10 mM Tris–HCl, 1% SDS, pH 8.0) at 55°C for 2 days, then washed with TE (10 mM Tris–HCl, 2 mM EDTA, pH 8.0) and treated by 0.75 μM phenyl-methyl-sulfonyl-fluoride (PMSF, Sigma-Aldrich) at 37°C for 10 min in order to inactivate residual proteinase activity. Finally, the plugs were washed with TE and stored in the same buffer at 4°C.

### In-gel enzyme digestion and labeling of yeast gDNA in agarose plugs

#### S1 nuclease digestion

Plugs were washed in S1 buffer (0.2 M NaCl, 50 mM Na-acetate, 1 mM ZnSO_4_, 0.5% glycerol, pH 4.5) three times for 30 min, then incubated with 500 U/ml S1 nuclease (Promega Biosciences Inc.) in S1 buffer for 1.5 h at 37°C (33).

#### Restriction endonuclease digestion

Plugs were washed in the appropriate 1× restriction buffer three times for 1 h and then further incubated with 150 U/ml restriction enzyme (Sfi I, Sma I, Mlu I, Pvu II or Stu I; Thermo Fisher Scientific) in 1× restriction buffer for 16 h at 30, 37 or 55°C according to the manufacturers’ recommendations.

#### In situ nick-translation using DNA polymerase I and biotinylated nucleotides

Plugs were used to incorporate biotin-dUTP by nick-translation performed under limiting conditions (‘limiting nick-translation’) that restricted incorporation to <200 bp regions (see Supplementary Figure S6). After washing in 1× DNA polymerase I buffer (50 mM Tris–HCl (pH7.5), 10 mM MgCl_2_, 1 mM DTT) three times for 20 min, the plugs were incubated with 150 U/ml DNA polymerase I (Thermo Fisher Scientific) in DNA polymerase I buffer containing 1 μM dNTP mix (1 μM biotin–dUTP, 1 μM dATP, 1 μM dCTP and 1 μM dGTP) and 5 μM ddNTP mix (5 μM ddATP, 5 μM ddTTP, 5 μM ddCTP and 5 μM ddGTP) for 30 min on ice to allow equilibration, then for 20 min at 37°C. The optimal dNTP/ddNTP ratio was determined by using a PCR product nicked at a specific site (Supplementary Figure S6). In the case of standard, ‘non-limiting nick-translation’, the ddNTP mix was omitted from the reaction.

#### Terminal deoxynucleotidyl transferase labeling

The reaction was performed using 260 U/ ml Terminal deoxynucleotidyl Transferase (TdT; Thermo Fisher Scientific) in its own buffer and 1 μM biotin-dUTP. The other conditions of the reaction were similar to those of the nick-translation.

#### Combined RNase treatment (RNase HI, A, H2)

Agarose plugs were digested with 12.5 U/plug RNase HI (Thermo Fisher Scientific) in 1× RNase HI buffer (20 mM Tris–HCl (pH 7.8), 40 mM KCl, 8 mM MgCl_2_, 1 mM DTT) at 37°C overnight, subsequently with 5 μl/plug of 10 mg/ml RNase A (Thermo Fisher Scientific) in TE for 1 h at room temperature then with 5 μl/plug of human RNase H2 enzyme (gift from Martin Reijns, University of Edinburgh) in RNase H2 buffer (60 mM KCl, 50 mM Tris–HCl pH 8, 10 mM MgCl_2_, 0.01% BSA, 0.01% Triton X-100) at 37°C overnight. Before each digestion, the plugs were equilibrated with the appropriate enzyme buffer three times for 50 min. All digestions were performed in 150 μl reaction volumes.

### Molecular combing

Molecular combing was performed on whole gDNA or on isolated chr XII, as described in (50). Genomic DNA, or chr XII isolated by CHEF, were embedded in agarose plugs and nick-labeled by biotinylated nucleotides (in limiting or non-limiting conditions, described above). chr XII was isolated from the plugs containing the whole genome by running this chromosome into a block of 0.5% LMP agarose inserted into a 1% standard agarose gel, then this block was cut out without EBr staining. To solubilize the whole genomic or chr XII DNA, 1.6 ml 0.1 M MES (pH 6.5) was added to each plug, incubated at 70°C for 20 min, then at 42°C for 10 min. The blocks were dissolved by 8 U Agarase (Thermo Fisher Scientific) treatment at 42°C overnight. In the case of combing of λ phage DNA, site-specific nicks were introduced by Nt.BbvCI nickase cutting 7 times in the phage genome (delimiting 306, 318, 614, 3977, 8013 and 12 451 bp fragments): 1.5 μg λ DNA was incubated with 50 U/ml Nt.BbvCI nickase (New England Biolabs) in 20 μl CutSmart buffer for 30 min at 37°C.

The DNA solutions were placed at room temperature and transferred to disposable reservoirs without pipetting. DNA combing was performed by the combing apparatus of Genomic Vision (France) according to the manufacturer's instructions using 22 × 22 mm vinylsilane coated coverslips (from the same source). After combing, the coverslips were glued to glass slides with cyanoacrylate glue. Nonspecific binding of the antibodies was blocked by incubation with 30 μl of 5% BSA/1× PBS/0.1% Triton X-100 (temporarily covering the combed sample with a clean coverslip) for 20 min in a humid chamber. The biotin molecules incorporated into the DNA were visualized by indirect immunofluorescent labeling with 1:60 diluted mouse anti-biotin as a primary antibody (Sigma-Aldrich). For R-loop detection, 33 μg/ml RNA:DNA hybrid specific S9.6 primary antibody (used as in (37); hybridoma from ATCC) was applied in 1% BSA/1× PBS/0.1% Triton X-100 for 45 min at room temperature in a humid chamber. After washing 3 times with 3 ml 1× PBS, twice with 30 μl PBS/0.1% Triton X-100 and then once with 30 μl PBS for 5 min, Alexa Fluor 647 conjugated goat anti-mouse antibody (Life Technologies) was used as a secondary antibody at a final concentration of 17 μg/ml in 1% BSA/1× PBS/0.1% Triton X-100, at room temperature in a humid chamber, for 45 min. In some experiments the enzymatically incorporated biotin and the RNA:DNA-hybrids were detected simultaneously on the same sample, using goat anti-mouse Abberior STAR 580 and streptavidin Abberior STAR RED as secondary reagents, respectively. After immunofluorescence labeling the coverslips were washed as before. DNA staining was performed by 30 μl YOYO-1 dye (Thermo Fisher Scientific) diluted 1:5000 in 0.1 M MES (pH 6.5) in a humid chamber, in the dark, for 20 min. The coverslips were covered by ProLong^®^ Gold Antifade using a clean coverslip and placed at 4°C overnight. Imaging was carried out in an Olympus FluoView 1000 confocal laser scanning microscope equipped with 488 and 633 nm lasers, using a 60× oil immersion oil objective.

### Genome-wide mapping of nicks (Nick ChIP-chip)

*Saccharomyces cerevisiae* (BY4741) cells were fixed in 1% formaldehyde (10 min, RT) and excess formaldehyde was quenched with 0.7 M glycine. Fixed cells were embedded into agarose plugs prepared according to standard protocols. Spheroplasts were obtained by lyticase digestion (Sigma-Aldrich) performed at 37°C for 3 h, followed by cell lysis in 0.43 M EDTA, 1% (v/v) Sarcosyl, 0.01 M Tris (pH 8) and 15 U/ml Proteinase K (55°C, 72 hours). At this point, one half of the sample was treated with nicking enzyme Nb.Bpu10I which recognizes CCTNA∧GC sites. Nickase plus and nickase minus samples were processed in parallel in the subsequent steps. Tagging of nicks was performed by incorporating biotin-dCTP/dUTP in the presence of chain terminator ddNTPs by the *E. coli* DNA polymerase I holoenzyme. Plugs were equilibrated in 2 ml of DNA polymerase I buffer for 3 × 50 min and then transferred to ice for 30 min in the nick-translating mix (consisting of 1× DNA polymerase I buffer, 5 μM of each ddNTPs, 1 μM of dATP, dGTP, biotin-dCTP, biotin-dUTP and 150 U/ml of DNA polymerase I). The reaction was initiated by transferring the tubes to 37°C for 30 min with gentle shaking, then stopped by washing the plugs in excessive amounts of 0.5 M EDTA. Agarose plugs were digested/solubilized with 2 U of β-agarase and sonicated (Bioruptor, Diagenode). Nucleic acids were purified by a PCR cleanup kit (Macherey-Nagel). RNA was digested with 10 μg/ml of RNase A in low-salt conditions (10 mM NaCl) at 37°C for 60 min. Immunoprecipitation of nicked DNA was performed according to the standard ChIP protocol for yeast using a monoclonal anti-biotin antibody (Sigma-Aldrich) coupled to Protein G coated Dynabeads (Thermo Fisher). Two-thirds of the immunoprecipitated DNA (IP) and an equal amount of input DNA were amplified by random primer extension followed by PCR, incorporating amino-allyl dUTP for subsequent dye coupling. Input and IP DNAs were labeled with Cy3 and Cy5, respectively, mixed in equal amounts, and hybridized to Agilent 4 × 44K whole genomic microarrays in 1× hybridization buffer for 16 h at 65°C. Microarray slides were scanned with an Axon 4000B scanner (using the GenePix5.1 software). In Figure 2, representative data of two (together with the nickase-digested sample of Supplementary Figure S8: three) biological replicates are shown.

Data were analyzed and nick peaks were called by the COCAS ChIP On Chip Analysis Suite (51). Genomic positions of annotated transcription units were obtained from the sacCer3 genomic assembly. The efficiency of cleavage/labeling was such that about 7% of the Nb.Bpu10I recognition sites were detected (Supplementary Figure S8A). Specificity of labeling was documented by the statistically highly significant coincidence of labeling with the nickase sites (Supplementary Figure S8B and C). The labeled sites in the nickase treated sample that coincide neither with the nickase recognition sites, nor with the endogenous nicks revealed in the sample without nickase treatment may be due to unrevealed endogenous nicks, or, less likely, off-target labeling.

### Enrichment analysis

To estimate the enrichment or depletion of nick peaks within annotation categories we chose to follow a previously described permutation test (52). Briefly, fold-change values were calculated by dividing the observed intercepting nucleotide occupancies of each annotation category with the mean occupancy of 1000 computer randomized (simulated) peaks. Random regions were generated with *shuffleBed* (53) with respect to the original peak sizes and chromosomal distribution. Significant difference was assigned with a two-tailed proportion test (http://www.socscistatistics.com/tests/ztest/).

### Signal density analysis

Mean RNAPII (54) around nicks (±1000 bp) or Nick ChIP-chip signal intensities around TSSs (±1500 bp) were calculated for 100/300 bp bins using Deeptools (55). The generated profiles were further processed and plotted in R. Random regions were generated with *shuffleBed* (53) with respect to the original peak sizes and chromosomal distributions. Significant difference was assigned with K–S tests or t-tests after checking the normality of distributions using the Shapiro-Wilk test.

### Gene expression correlation

For each nick (peak) position (*n* = 215) we assigned the closest protein coding gene with *closestBed* (53) and their expression values were plotted as a boxplot. We randomly sampled 215 genes from the total gene pool 1,000 times and compared their expression levels with that of the nick-associated genes. Overlapping genes were considered based on their average expression level. Significant difference was assigned with the Mann-Whitney test; *P*-values were corrected with the Benjamini & Hochberg method. Expression values for wild-type *S. cerevisiae* genes were obtained from GEO (GSE98435) (56). Correlation with RNAP II chromosomal binding was analyzed using publicly available ChIP-Chip datasets downloaded from GEO (GSE6293) (54).

### Mapping of S1-sensitive sites by Southern-blot analyses

Log-phase *S. cerevisiae* cells were fixed in 1% HCHO as described above and used to prepare agarose embedded spheroplasts. The plugs were digested with S1 nuclease and rare cutting restriction enzymes (Sfi I, Sma I, Mlu I or Hind III, single-cutter restriction enzymes in the rDNA units), applied in a sequence indicated in Figures 3–5, S12 and S14, and the restriction fragments were separated by conventional or urea/heat-agarose gel electrophoresis. The gels were blotted to Hybond N^+^ membrane (Amersham GE Healthcare Life Science) by a vacuum blotter (Bio-Rad Model 785).

A 1405 bp ds fragment covering the rDNA region shown in Figure 4A, amplified using the primers (pRDs (5′-GGG GAT CGA AGA TGA TCA GA-3′; Integrated DNA Technologies (IDT)) and pRDas (5′-GAA AAG GCC AGC AAT TTC AA-3′; IDT), served as the template for the preparation of single-stranded probes. First, linear amplification was performed using 2.5 U Taq polymerase (Thermo Fisher Scientific), in 50 μl of 1× reaction buffer (10 mM Tris–HCl, 50 mM KCl, 0.08% Nonidet P-40, pH 8.8) containing 3 mM MgCl_2_, 200 ng template DNA, 20 pmol of either pRDs (sense) or pRDas (antisense) primer, dATP, dTTP, dGTP and dCTP at 0.25 mM concentration (from Promega Life Science, Madison, USA). The probes were purified on Sephadex G-25 spun columns for random primer labeling using [α^32^P]-dCTP (6000 Ci/mmol, 10 mCi/ml; Institute of Isotopes LTD, Budapest), as described earlier (57). The probes were denatured for 10 min at 100°C and kept on ice for 5 min before hybridization. Note that a small amount of complementary strand (derived from the co-purified original template DNA) was also present in the single stranded probes. The radioactive signal was captured by Phospho-screen (Kodak) and was visualized by a BIO-RAD Phospho-Imager.

For the estimation of ds or ss fragment sizes in Southern blot experiments calibration curves were constructed for ds and ss DNA molecules, based on ds DNA ladders and mixtures of denatured rDNA PCR products, respectively.

### Reverse Southwestern blot

rSW blot (58) was carried out following a protocol developed by us, that is described below and is explained in Supplementary Figure S15. Plugs containing *S. cerevisiae* gDNA after limiting *in situ* nick-translation with DNA polymerase I (see above) were digested in lysing solution (0.5 mg/ml Proteinase K, 1% SDS, 0.5 M EDTA, 10 mM Tris; pH 8.0) at 54°C for 30 min, then washed with TE and treated with 0.25 mM PMSF at 37°C for 10 min to inactivate residual proteinase activity. Finally, the plugs were digested with the rare cutting restriction endonucleases Sfi I or Sma I. The DNA fragments were separated on 1% agarose gel, and the 9.1 kb rDNA units were carefully cut out from the gel and were further digested for 16 h at 37°C with the mixture of two different restriction endonucleases (Pvu II *+* Stu I). The restriction fragments of rDNA units were separated on 1.2% agarose gels and vacuum-blotted on Immobilion-P Transfer Membrane (PVDF, 0.45 μm, Millipore). These were prehybridized for 1 hour at room temperature in 5 ml prehybridization solution (1% BSA, 0.2% Tween-20/1× PBS), then incubated with mouse anti-biotin primary antibody at a dilution of 1:1000 in 5 ml hybridization solution for 16 hours at 4°C. After washing with 0.2% Tween-20/1× PBS for 5 × 5 min, the membranes were incubated with goat anti-mouse IgG antibody conjugated with horseradish peroxidase (dilution 1:2500) at room temperature, for 1.5 h. We found that the choice of the membrane and of the blocking agent were crucial. The use of milk powder as a blocking agent is not satisfactory in the case of biotinylated targets, because its biotin content gives a strong background, therefore BSA is recommended as a blocking agent. The signal was detected by chemiluminescence (KODAK Medical X-ray Processor). The dynamic detection range of the method was calibrated with biotinylated PCR products (Supplementary Figure S16). When the membranes were stripped and re-probed for the detection of R-loops in rDNA units with the RNA:DNA hybrid specific S9.6 antibody (used at 1 μg/ml concentration), the membranes were washed four times for 5 min each in 0.2% Tween-20/1× PBS, then incubated in stripping buffer (62.5 mM Tris base, 2% SDS, 0.7% 2-mercaptoethanol, pH 6.8) for 30 min at 50°C, then washed six times for 5 min in 0.2% Tween-20/1× PBS. Blots were evaluated by the ImageJ software. For quantification, the biotin signal intensities of the fragments relative to their EBr signal intensities were calculated. To compare the intensities in different yeast cells, the calculated biotin/EBr ratios were normalized to the band having the highest ratio on the gel (taken as 1.0 on the Y-axis of the histogram). Since the ratios are proportional to the incidence of nicks in a given amount of DNA (EBr signal) the observed changes are independent of differential loading and/or the copy number of rDNA units.

## RESULTS

### Endogenous ss breaks in *S. cerevisiae* gDNA are revealed by molecular combing and gel electrophoretic analyses

**Figure 1.**
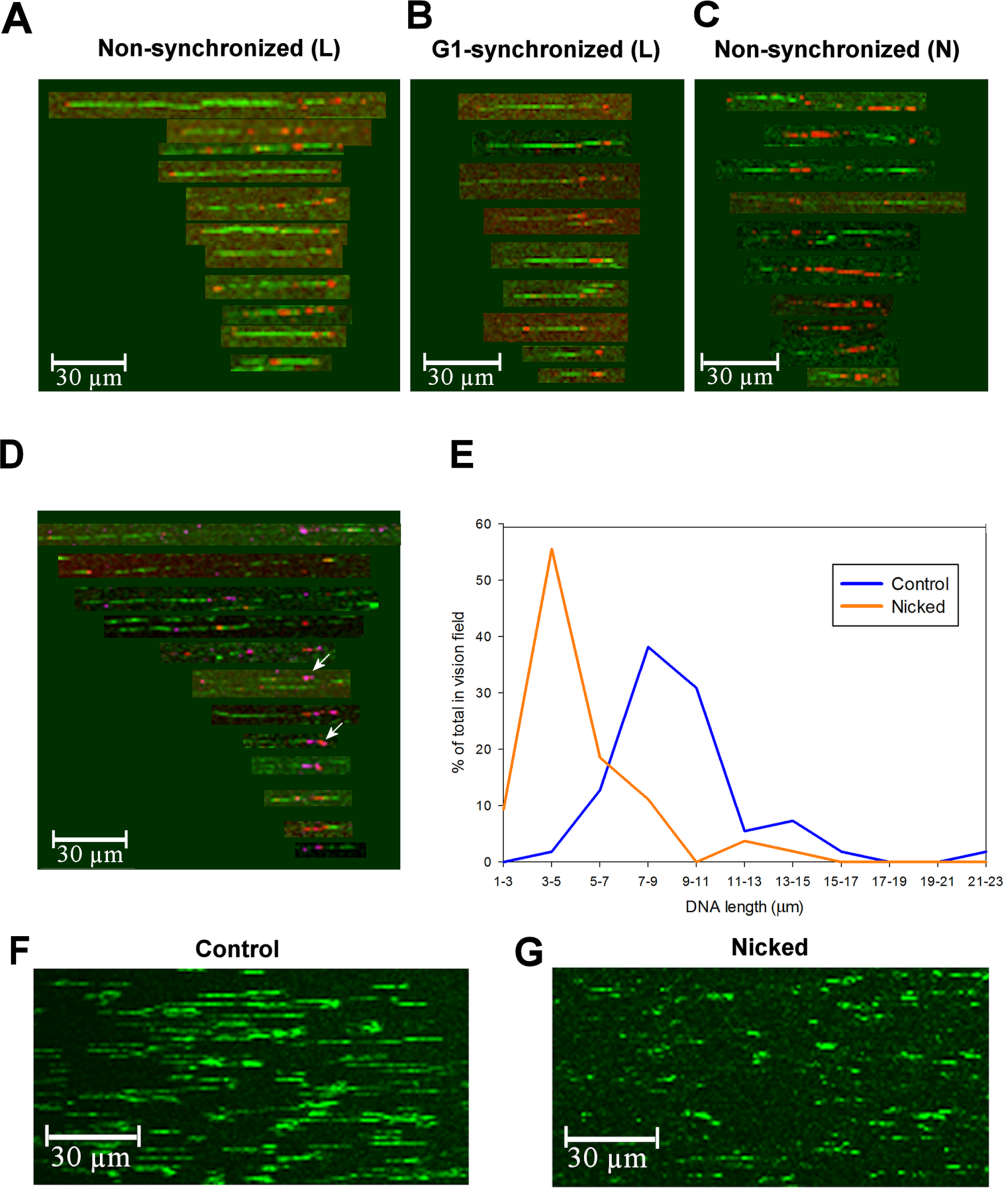
Breakage of *S. cerevisiae* gDNA and λ DNA at preformed ss nicks upon molecular combing. (**A-C**) Molecular combing of nick-translated gDNA from *S. cerevisiae*. Biotinylated nucleotides were incorporated by nick-translation conducted either in limiting (L), or non-limiting/standard conditions (N), into agarose-embedded gDNA of unperturbed, non-synchronized (A and C), or G1-synchronized (B) BY4741 cells. Biotin was detected by AlexaFluor 647-conjugated anti-biotin antibody (red) and DNA molecules were stained with YOYO-1 (green). Panel **D** shows examples of co-localization of nicks labeled with TdT (magenta) and R-loops labeled with the RNA:DNA hybrid specific S9.6 antibody (red), when both entities were visualized in the same sample. The percentage of co-labeled spots was estimated ∼10% of all nick-related DNA associated spots. Arrows indicate examples of co-localization. (**E–G**) Molecular combing of λ DNA. Representative images of YOYO-1 stained (green) control (F) and Nt.BbvCI nickase-treated (G) λ DNA. The size distribution histograms of combed DNA molecules before (blue) and after (orange) nickase treatment are shown in panel E. The full length intact ds λ DNA (48.5 kb) corresponds to 16.2 μm (calculated with 3 bp/nm helical repeat length), i.e. the majority of λ DNA molecules were fragmented after combing alone. Images of DNA fibers were assembled from the fields-of-view analyzed, except for panels F and G which show the original fields-of-view. For statistics see Supplementary Tables S1–S5.

The purpose of the experiments described below was to detect endogenous DNA breaks via their free 3′OH groups and to determine whether they belong to ss or ds termini. DNA strand breaks with free 3′OH could be visualized in molecular combing experiments when biotinylated nucleotides were incorporated into agarose embedded deproteinized gDNA by limited nick-translation (mixing terminator nucleotides with dNTPs; see Materials and Methods). As shown in Figure 1, the combed DNA molecules were similar to or larger than the combed λ phage DNA (Figure 1A, B versus F), and often carried the 3′-label at the fragment ends, sometimes on both sides (Figure 1A). Similar labeling was detected in α-factor-synchronized G1 cells (Figure 1B), suggesting that endogenous nicks can occur independently of DNA replication. The free 3′OHs revealed by labeling could be either (i) part of preexisiting ds fragment ends, or (ii) belong to preexisiting single-strand (ss) discontinuities, likely nicks, which could serve as predilection points for mechanical breakage of the intact strand during solubilization of the plugs and/or combing. In a model experiment, the average size of combed λ phage DNA nicked at specific sites by a nickase enzyme (see Materials and Methods) was reduced relative to the length of the phage DNA molecules without nickase treatment (Figure 1E–G). Therefore, it is likely that the preferential end-labeling of the ds fragments obtained upon combing was the consequence of mechanical breakage upon solubilization or combing, at pre-existing, labeled nicks, even though no pipetting or mechanical shearing were applied in the whole protocol. The presence of nicks in the agarose-embedded DNA was demonstrated by standard nick-translation experiments (in the absence of terminator nucleotides) where labeled DNA stretches encompassing a few kb were visualized (Figure 1C). Such long stretches could become labeled by the 5′-3′ polymerase activity of the DNA polymerase I (involving either nick-translation or strand-displacement). Incorporation of the biotinylated nucleotide in the case of ds breaks with overhanging 5′ end would result in spot-like end-labeling due to the limited length of the overhang at a ds break (in contrast with two, non-apposed ss breaks). (See Supplementary Tables S1-S5 for statistical analysis of the combing experiments.) In order to exclude the possibility that the labeling originated from free 3′OH of RNA (see (59)) rather than DNA molecules, terminal transferase (TdT) treatment was followed by combined RNase digestion applying subsequently RNase A, RNase HI and human RNase H2 treatments (based on (60–62)). Note that this schedule should preclude labeling of RNase-induced nicks at misincorporated ribonucleotides. RNase H enzymes specifically degrade the RNA portion of the RNA:DNA hybrids, so the RNA-primed fragments should diffuse away from the sites of labeling. Importantly, biotinylated nucleotides incorporated by TdT were not removed by RNase treatment (Supplementary Figure S1A and B). The efficiency of RNase treatment was indicated by the fragmentation of the DNA likely due to unrepaired, misincorporated ribonucleotides in the genome (63–65) and by the disappearance of S9.6 staining (21,37) of the R-loops (Supplementary Figure S1C and D) that also appeared frequently at the ends of the combed fragments (Supplementary Table S2). Remarkably, coinciding R-loop and nick signals were also detected upon co-labeling (Figure 1D), suggesting that these two features are occasionally positioned within ∼1 kb from each-other (in view of the resolution of the microscope). In summary, the DNA breaks giving rise to loop-size fragments in combing experiments may represent endogenous nicks with free 3′OH ends.

In order to determine the overall incidence of nicks along the genome and assess their relative localization on the two strands, we performed gel electrophoretic analyses by FIGE (Supplementary Figures S2 and S3, for *S. cerevisiae* and *S. pombe*, respectively). Treatment of the DNA with S1 nuclease revealed S1-sensitive regions delimiting ∼100 kb intervals on the ds DNA (Supplementary Figure S2A). These may include both endogenous nicks and alternative DNA structures containing ss regions (like hairpins, R-loops, G-quadruplexes, etc.). However, the ss fragments observed after urea/heat (and also alkaline) denaturation offer a *bona fide* indication of DNA breaks (see Supplementary Figure S2B). Note that urea/heat-denaturation ((57); explained in Supplementary Figure S4) is a method which avoids the hydrolysis at misincorporated ribonucleotides that may happen in alkaline gels (64,66). The ss size of the DNA denatured and analyzed after S1 digestion was smaller than without digestion, suggesting that mainly nicks arranged on the two strands in a non-apposed manner are the sites of S1 digestion (compare Supplementary Figure S2B lanes 4–7 with the schemes of Supplementary Figure S2C and D). The size of the urea/heat-denatured ss fragments was very similar to that of denatured T4 phage DNA (∼169 kb; data not shown).

Supplementary Figure S5 shows that the average ss fragment size assessed by S1 digestion or urea/heat-denaturation was not affected by the addition of ethidium bromide (EBr) that relaxes or overwinds DNA depending on its concentration (37,67,68). If nicks on the opposite strands were separated by short (< 25 bp) stretches of DNA, disassembly into loop-size ss fragments upon denaturation would readily occur at ≤80°C (see the Supplementary Figure S5 legend for the calculation), in sharp contrast to what was observed. In line with the conclusion that nicks far away from each other on the complementary strands are responsible for the appearance of the ∼150 kb ss fragments upon denaturation, the 2 μ plasmid that carried no nicks (Supplementary Figure S2B, lanes 2–3) was denatured at a very similar EBr concentration as gDNA (Supplementary Figure S5B).

Collectively, the above data confirm that the nicks on the complementary strands are arranged in a non-apposed manner, i.e. they represent nicks rather than ds breaks, in agreement with the results of the combing experiments. They also suggest that the emergence of the breaks is not related to an abrupt redistribution of twist and writhe along the loops, which is likely to occur upon lysis/deproteinization (69).

Since shearing-related mechanistic origins of a post-lysis generation of the breakages were excluded in our earlier reports (33,36), the discontinuities observed here appear to be pre-exisisting, endogenous breaks, generated *in vivo*. The incidence of labeled sites in the combing experiments was around one in 70–100 kb DNA, so it is plausible to conclude that most of the breaks detected in the gel electrophoretic experiments (i.e. ∼1 in 100 kb) possess free 3′OH ends.

### Genome-wide distribution of endogenous nicks: microarray analyses suggest RNAP II promoter-proximal enrichment

**Figure 2.**
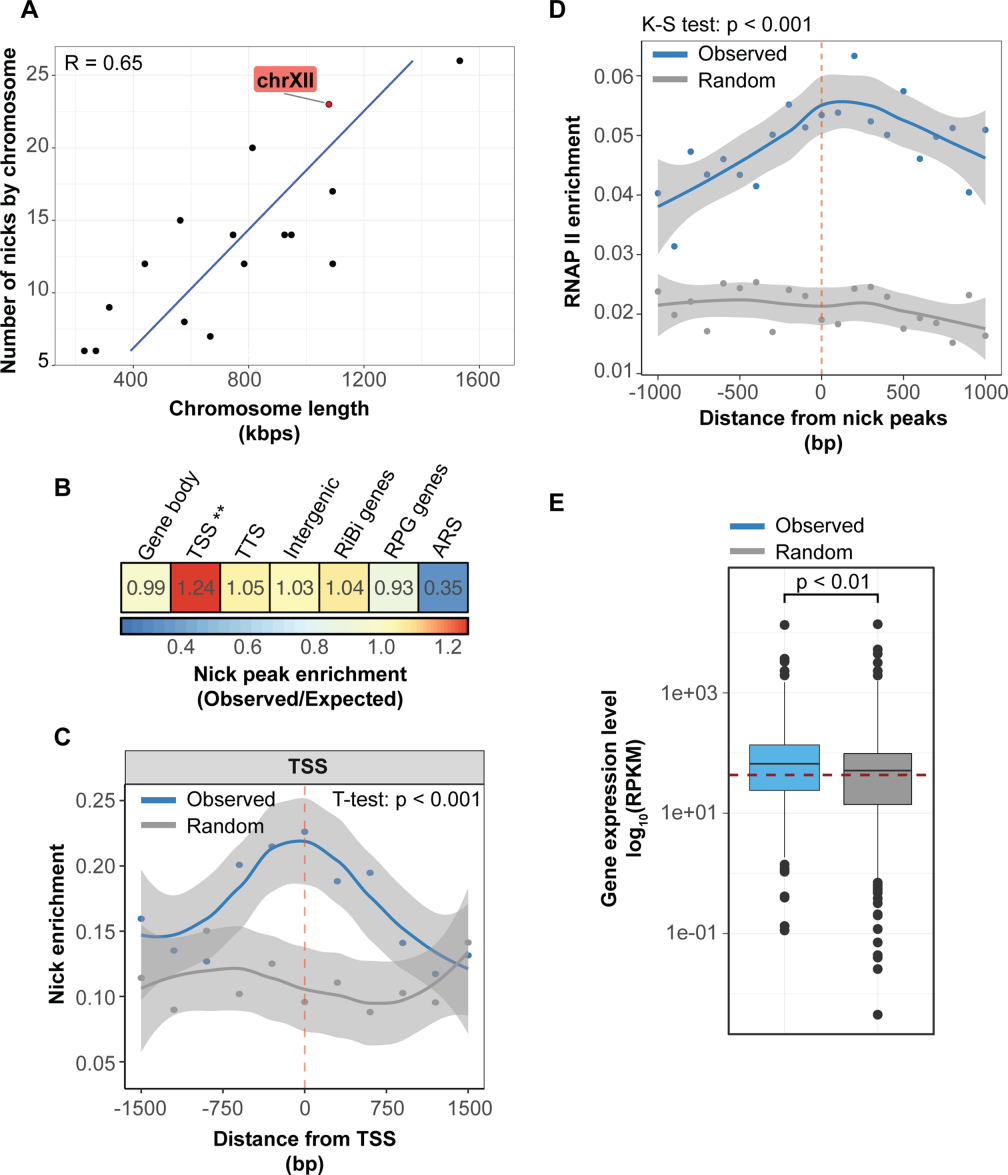
Distribution of nicks with free 3′OH ends along the gDNA of *S. cerevisiae*. (**A**) The frequency of nicks positively correlates with chromosome length. The number of nicks determined in one of the microarray experiments is shown for the *S. cerevisiae* chromosomes. R: Pearson correlation coefficient. Dots indicate the number of nicks per chromosome, chr XII is highlighted. (**B**) Distribution of nicks (peaks) within the functional categories of the *S. cerevisiae* genome (TSS: transcription start sites; TTS: transcription termination sites; RiBi: ribosome biogenesis genes; RPG: ribosomal protein genes; ARS: autonomous replicating sequences). Heatmap shows the ratio of overlapping nucleotides between observed and randomly permutated nick positions within the indicated genomic category. Significant difference was assigned with the two-tailed proportion test (**P < 0.001). (**C**) Nick signals are preferentially enriched at transcription start sites (TSSs; *n* (total gene pool) = 6664). The average binding intensity of Nick ChIP-chip signal is shown as a scatterplot for observed (blue) and randomly permutated (gray) nick peaks (±1500 base pairs). Lines represent the smoothed mean after loess normalization. Faded area shows the 95% confidence interval for predictions from the loess model. Significant difference was assigned using the two-tailed Student's *t*-test. (**D**) RNAP II tends to accumulate over nick peaks in *S. cerevisiae*. The average binding intensity of RNAP II is shown for observed (blue) and randomly permutated (gray) nick peaks (+/- 1000 base pairs). Lines represent the smoothed means after loess normalization. Faded area shows the 95% confidence interval for predictions from the loess model. Significant difference was assigned using the Kolmogorov–Smirnov (K–S) test (two-tailed). (**E**) Nick-associated genes show elevated mRNA expression. Proportional box plots show the expression levels (RPKM) of nick-associated genes (blue; *n* = 215) and of random genes (gray; *n* = 215). Red dashed line represents the genome-wide median mRNA levels. Significant difference was assigned using the Mann–Whitney rank sum test.

To find out if the nicks are scattered randomly or accumulate at particular sites over the *S. cerevisiae* genome, we performed microarray experiments (Figure 2), using normally cycling, non-synchronized cells. As most of the nicks carry free 3′OH ends (see above), nick-translation could be used to label these breaks. After deproteinization of the agarose-embedded and formaldehyde-fixed spheroplasts, these sites were nick-labeled with biotinylated nucleotides under limiting conditions (see Supplementary Figure S6 for optimization of these conditions). Subsequently, the sonicated DNA samples were immunoprecipitated, amplified and hybridized onto tiling microarrays covering the non-repetitive regions (i.e. excluding rDNA, telomeric regions, tRNA genes and retrotransposons) of the *S. cerevisiae* genome. As Figure 2A shows, the overall frequency of nick overlapping sequences detected by the array was proportional to the length of the chromosomes. The incidence of the endogenous nicks was ∼1/70 kb (calculation based on Figure 2A). Remarkably, the nicks accumulate at the RNAP II transcription start sites (TSSs; Figure 2B, C and Supplementary Figure S7A) as opposed to the transcription termination sites (TTSs; Supplementary Figure S7B), the autonomously replicating sequences (ARSs), and other genetic regions (Figure 2B). RNAP II distribution (data from (54)) peaks around nicks (Figure 2D) and the incidence of nicks significantly correlates with gene activity (Figure 2E; and Supplementary Figure S7A). To assess the sensitivity and accuracy of the whole procedure, some of the samples were also digested with a frequent cutter nickase enzyme of known specificity (Supplementary Figure S8A–C; see Materials and Methods). Annotation of the nicked promoters in different experimental samples shows partially overlapping sets (Supplementary Figure S8D). R-loops, that can be detected by the S9.6 antibody, were absent from the TSSs and accumulated at the TTSs (unpublished data in line with (70,71)), so most probably we deteced true nicks at the TSSs. Similar, TSS-proximal accumulation of nicks was observed using wild-type cells at different metabolic conditions and alpha factor synchronized (G1) *bar1Δ* cells (Supplementary Figure S8D and data not shown). The results of the microarray analyses were confirmed in an independent ‘chip-on-beads’ experiment according to (72,73): We demonstrated enrichment of Ser5-phosphorylated (i.e. initiating and elongating; see (74)) RNAP II in the <500 bp vicinity of nicks, whereas no such an accumulation of the Ser2-phosphorylated, elongating and terminating species was observed (Supplementary Figure S9). The majority of initiating RNAP II molecules detected in the assay are considered primarily gDNA-bound.

Collectively, these data suggest that endogenous nicks are distributed along the chromosomes in a non-random manner, accumulating at RNAP II promoters, strongly suggesting that they are transcription- rather than replication-related.

### Distribution of endogenous nicks within chromosome XII of *S. cerevisiae* defines ∼100 kb loops

**Figure 3.**
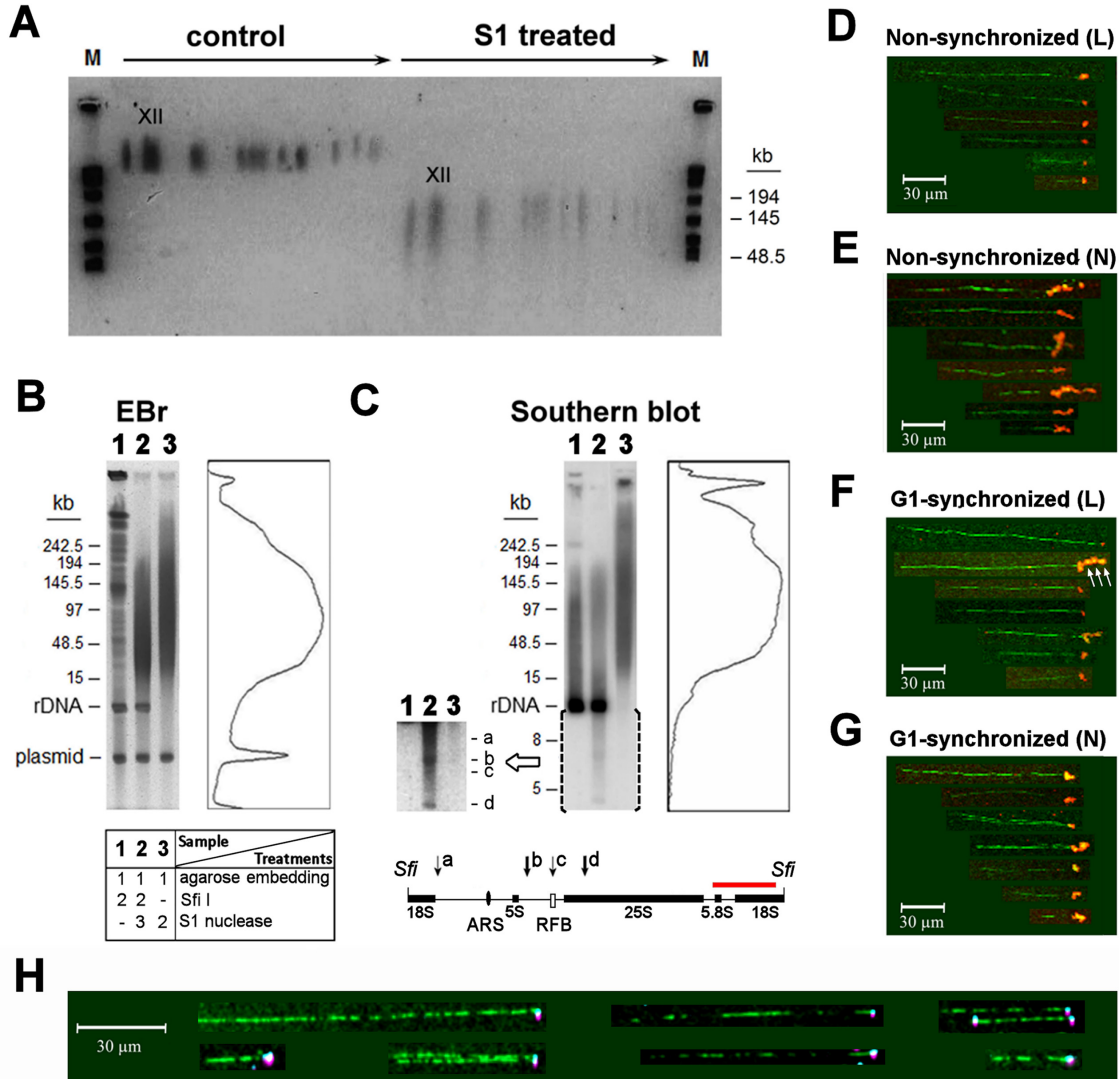
Endogenous nicks delimit loop-size intervals in chromosome XII of *S. cerevisiae*. (**A**) Two-dimensional gel electrophoretic analysis of S1 sensitive sites in *S. cerevisiae* chromosomes. Deproteinized chromosomal DNA molecules resolved by CHEF (first dimension) were digested with S1 nuclease (or treated with S1 buffer as a control), then further separated in the second dimension by FIGE. The arrows point from the large to the small chromosomes. The order of chromosomes from left to right is IV, XII (marked), VII/XV, XIII/XVI, II, XIV, X, XI, V/VIII, IX, III, VI, I. (B, C) CHEF and Southern blot analysis of rDNA specific S1 sensitive sites in the deproteinized DNA of agarose embedded WDHY199 spheroplasts. (**B)** EBr-stained CHEF gel after electrophoresis of the Sfi I and/or S1 digested samples in a representative experiment using the same strain. Lanes: 1. Sfi I digestion; 2. Sfi I and S1 digestion; 3. S1 digestion. The densitometric size distribution of gDNA fragments after S1 nuclease digestion (in lane 3) is shown right to the gel. The table underneath the agarose gel image shows the order of treatments. (**C**) Southern hybridization using an rDNA specific probe. The bracketed section left to the blot shows an enhanced exposure. The sketch below the blot shows positions of rDNA fragments a-d on the rDNA map. These fragments were derived by S1 digestion of the Sfi I digest (panel C, lane 2), as detected by the probe (red). The size distribution of rDNA after S1 nuclease digestion (panel C, lane 3) is shown right to the blot. The rDNA fragments appeared as a 9.1 kb band upon Sfi I digestion that cuts once in every unit (panels 3B and C, lanes 1–2). The 2-micron plasmid, a small multi-copy selfish DNA element of the strain ((134); labeled as ‘plasmid’) served as an internal negative control confirming that the hybridization signals were rDNA specific. (D–G) Molecular combing of nick-translated chr XII. Biotinylated nucleotides were incorporated by limiting (L), or standard/non-limiting (N) nick-translation into the gDNA of agarose embedded non-synchronized (panels D and E), or G1-synchronized (panels F and G) BY4741 cells, before separation of the chromosomes by CHEF. (**D**) chr XII from non-synchronized cells after limiting nick-translation; (**E**) chr XII from non-synchronized cells, after non-limiting/standard nick-translation; (**F**) chr XII from G1-synchronized cells, after limiting nick-translation (white arrows point to several juxtaposed labeled rDNA units); (**G**) chr XII from G1-synchronized cells after non-limiting, standard nick-translation. Biotin label was visualised using AlexaFluor 647-conjugated anti-biotin antibody (red). Note that the stretches of labeled DNA are folded to form large, bright spots. (**H**) Labeling of R-loops on combed *S. cerevisiae* chr XII DNA. The RNA:DNA-hybrids were detected by the S9.6 antibody (pink), and the displaced, ss DNA was stained using Cy3B-conjugated SSBP (cyan; prepared and labeled as described previously (75)). DNA molecules were stained with YOYO-1 (green). Images were assembled from the fields-of-view analyzed. For statistics see Supplementary Tables S1, S3 and S5.

In agreement with the microarray data, the overall manifestation of endogenous nicks detected by two dimensional gel ectrophoresis (described in Supplementary Figure S10) was similar for the 16 *S. cerevisiae* chromosomes (Figure 3A). Moreover, the nick distribution within the three *S. pombe* chromosomes, as revealed either by S1 digestion or urea/heat-denaturation, was also similar (Supplementary Figure S11), indicating that the nick-generating mechanisms may be closely related in these two genetically distant model organisms. Interestingly, chr XII (carrying the rDNA cluster) also exhibited a similar fragmentation pattern, suggesting that sequence-unrelated factors determine the incidence of ss breaks. Indeed, the DNA fragments detected on Southern blots by an rDNA specific probe showed a size distribution similar to that of the total gDNA (Figure 3B and C). Sizing was performed using CHEF, yielding a more accurate picture of fragment length distribution than FIGE (used in (25,26,33,36–38)). The ds fragment size range was between 20 and 200 kb, with the median of distribution at ∼100 kb. Thus, every ∼10th of rDNA units of identical sequence harbors nicks on any of the DNA strands. Collectively, these data suggest that sequence-related factors alone do not determine where the nicks are generated.

Endogenous nicks could also be detected in chr XII by molecular combing, as shown in Figure 3D–H. The nicks, revealed by enzymatic labeling of the free 3′OHs, were present in the DNA of G1-synchronized cells, excluding DNA replication as a main source of nick formation. In agreement with the results of whole genome combing experiments (Figure 1C), the stretches of labeled DNA observed in standard, non-limiting nick-translation conditions indicated that ss discontinuities were detected (Figure 3E and G). The quantitative evaluation of the labeling frequencies in the case of gDNA and chr XII is presented in Tables S1, S3 and S5. Remarkably, very few combed chr XII fragments carried label on both ends (Supplementary Table S5). Molecules carrying several labeled sites separated from each-other at ∼10 kb distances were also observed in the case of limiting nick-translation (arrows in Figure 3F). Nicks appear to be less frequent in chr XII than in the whole gDNA, perhaps because smaller fragments bordered by neighboring nicks within a single rDNA unit (see Figure 6) have been lost during combing. We also found that DNA molecules derived from G1 synchronized cells contained nick-labeled spots less frequently than those of asynchronous/non-perturbed cells (Supplementary Table S3), as a result of the absence of Okazaki fragments in G1. Surprisingly, S9.6 labeling was also prevalent at the fragment ends of chr XII (Figure 3H). The identity of R-loops was further confirmed by their co-localization with recombinant, bacterial single-strand binding protein (SSBP, (75); Figure 3H).

### Mapping of the nicks within the rDNA units of chromosome XII demonstrates accumulation at discrete RNAP II-related sites

**Figure 4.**
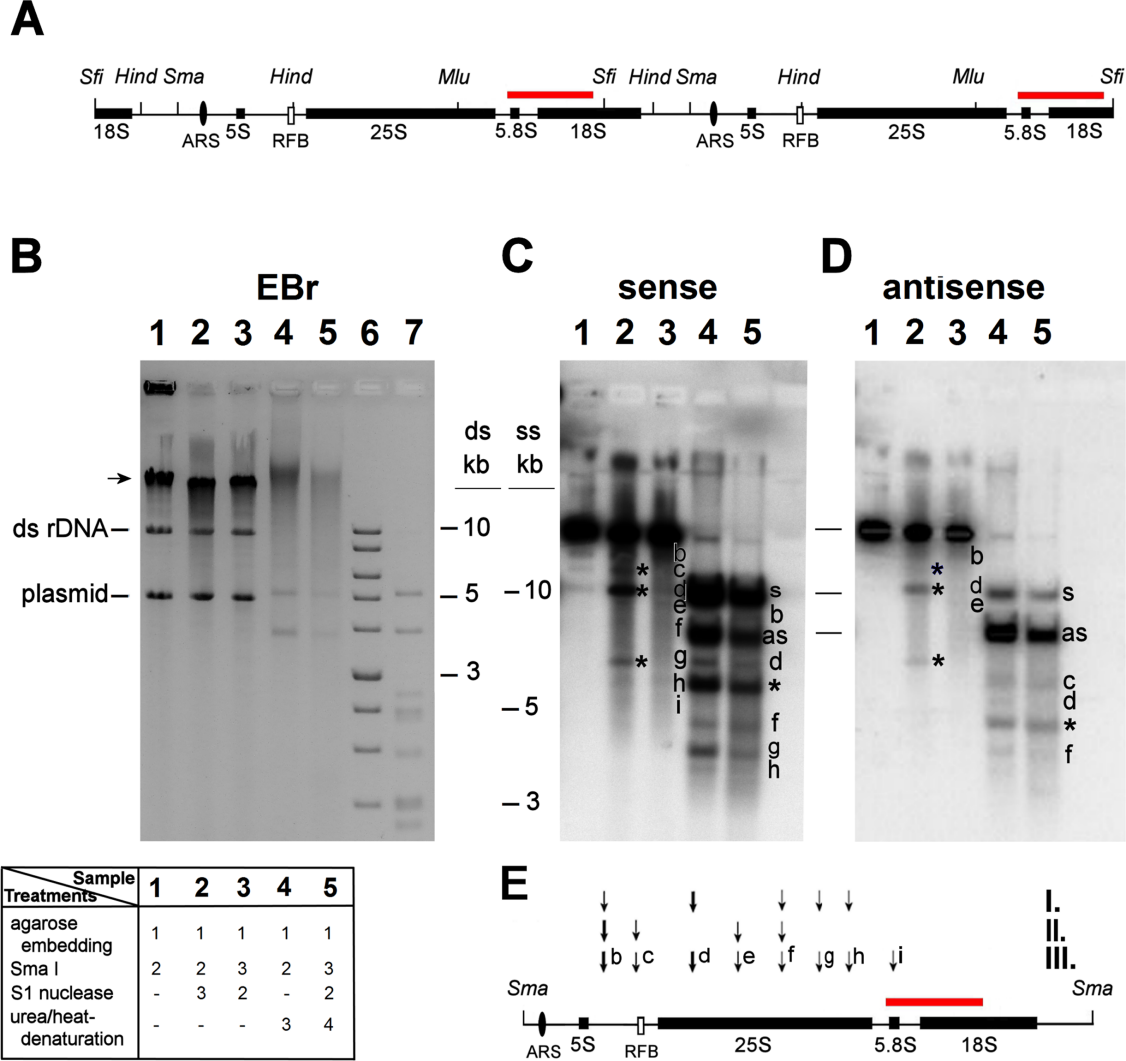
Nicks accumulate at discrete sites within the rDNA units. Deproteinized WDHY199 spheroplasts were analyzed by Southern blotting using strand specific rDNA probes. (**A**) Restriction map of two contiguous rDNA units. The black boxes indicate the 25S, 18S, 5.8S and 5S exons. ARS and RFB are illustrated by a black oval and a white rectangle, respectively. The hybridization position of the Southern probes is indicated by a red bar. Sfi: Sfi I; Hind: Hind III; Sma: Sma I; Mlu: Mlu I. Note that Sfi I, Sma I and Mlu I are rare cutting restriction endonucleases. (**B**) EBr stained gel containing gDNA after: Sma I cleavage (lane 1); Sma I cleavage followed by S1 nuclease digestion (2); S1 nuclease digestion followed by Sma I cleavage (3); Sma I cleavage followed by urea/heat-denaturation (4); S1 nuclease digestion, then Sma I cleavage, then urea/heat-denaturation (5). The order of treatments is summarized by numbers in the table underneath the agarose gel image. Lanes 6 and 7 contain non-denatured and denatured 1 kb marker, respectively. Arrow: compression zone; plasmid: 2-micron plasmid (134); ds rDNA: 9.1 kb non-denatured rDNA units. (C and D) Southern hybridizations with sense (**C**), and antisense (**D**) strand-specific probes. ‘s’ and ‘as’ indicate the separately migrating 9.1 kb sense and antisense strands of denatured rDNA units, respectively. Note that the difference in electrophoretic migration between the two strands of same size is due to their different base composition which leads to different overall conformational characteristics even in the presence of the denaturing agent (see (57)). The signal intensities were consistently stronger in the case of the electrophoresed ss than ds DNA, possibly because hybridization of the ss probe to ss target sequences is more efficient than to ds target sequences. ‘b-i’: fragments derived from endogenous nicks revealed by S1 nuclease digestion or urea/heat-treatment; *: artefactual fragments generated by the nicking activity of Sma I. (See Supplementary Figure S13 for the consequences of ‘off-target’ nicking.) (**E**) The scheme summarizes the location of nicks at one rDNA unit. The arrows indicate the positions of nicks (‘b-i’) mapped in the rDNA. I.: nicks detected with the sense strand-specific probe in denatured, ss rDNA samples (lanes 4 and 5); II.: nicks detected with the antisense strand-specific probe in denatured, ss rDNA samples (lanes 4 and 5); III.: nicks converted to ds breaks with S1 nuclease, detected with either sense or antisense strand-specific probes in non-denatured, ds rDNA samples (lane 3). One representative blot of at least three independent experiments is shown.

To map the arrangement of nicks within the rDNA cluster, we employed restriction endonucleases that cut only once in each rDNA unit (Figure 4A) followed by Southern blotting. The cleavages in the other parts of the gDNA produced high molecular weight fragments that remained in the compression zone of the gels (e.g. Figure 4B lane 1). The nicked sites were determined after S1 nuclease digestion or urea/heat-denaturation, when the rDNA units were cut out by Sfi I (Figure 3B), Sma I (Figure 4 and Supplementary Figure S12), and Mlu I or Hind III (Supplementary Figure S12). The nicks present in the rDNA units gave rise to discrete bands (Figure 4C and D, lanes 2 and 3; summarized in Figure 4E). Many restriction endonucleases may artefactually nick the DNA at sequences differing from the recognition site with only one base pair (19,20). To distinguish the genuine endogenous nicks from off-target nicking by restriction enzymes, the S1 nuclease and the restriction enzymes were applied in alternating sequence (for an illustration of the rationale see Supplementary Figure S13). Based on the comparison of the two cleavage patterns (exemplified by lanes 2 and 3 of Figure 4C and D), off-target nicks (labeled by an asterisk in Figure 4 and Supplementary Figure S12) created by restriction endonucleases were eliminated from the maps of endogenous nicks. This problem was avoided when Sfi I was used, since this enzyme generated no artefactual nicks within the rDNA units (see Figure 3B-C and compare lanes 2 and 3 in Supplementary Figure S14). The arrangement of nicks in each strand of the rDNA was also investigated by using strand-specific ss probes (Figure 4 and Supplementary Figures S12 and S14) following urea/heat-denaturation of rDNA units into differentially migrating complementary strands (as in (57,76)). Nicks that were mapped by S1 nuclease (i.e. when restriction enzyme digestion was preceded by S1 treatment), and those that were also observed in the urea/heat-denaturing electrophoretic experiments, were considered genuine, preformed nicks, not caused by restriction enzyme digestion, or by locally denatured regions of the ds DNA. The positions of endogenous breaks are represented on the rDNA maps of Figures 4E and 6, and in Supplementary Figures S12 and S14. Some nicks were detected both on the sense and antisense strands by single strand specific probes, although ds breaks were not detected within the rDNA units without S1 digestion (Figure 4C and D, fragment ‘f’; compare lane 1 with lanes 4 and 5). Such nicks may have occurred either in one or in the other strand in the different rDNA units, or, alternatively, arranged within the affected units close enough to be mapped to the same position using the ss probes, but distant enough so that the two strands can be kept together.

Taken together, these data show that endogenous nicks accumulate at discrete sites along the rDNA units, except for a ∼2 kb region encompassing the ARS (see Figure 6). In line with the reduced incidence of nicks in the ARS-containing region, the position of the ss breaks was indistinguishable when α-factor-, or nocodazole-synchronized, G1 and G2/M phase arrested cells, respectively, were studied (Supplementary Figure S14), suggesting that the endogenous nicks occur independently from, and persist through DNA replication.

The arrangement of nicks was insensitive to RNase HI or RNase A treatment of the plugs (Supplementary Figure S12 and data not shown), indicating that cleavages by S1 nuclease at the ss DNA of the R-loops in rDNA (77,78) do not contribute to the cleavage patterns observed in Southern hybridisations. In support of this conclusion, the localization of R-loops and nicks along the rDNA unit diverged from each-other in IGS1 and IGS2 regions (see below).

**Figure 5.**
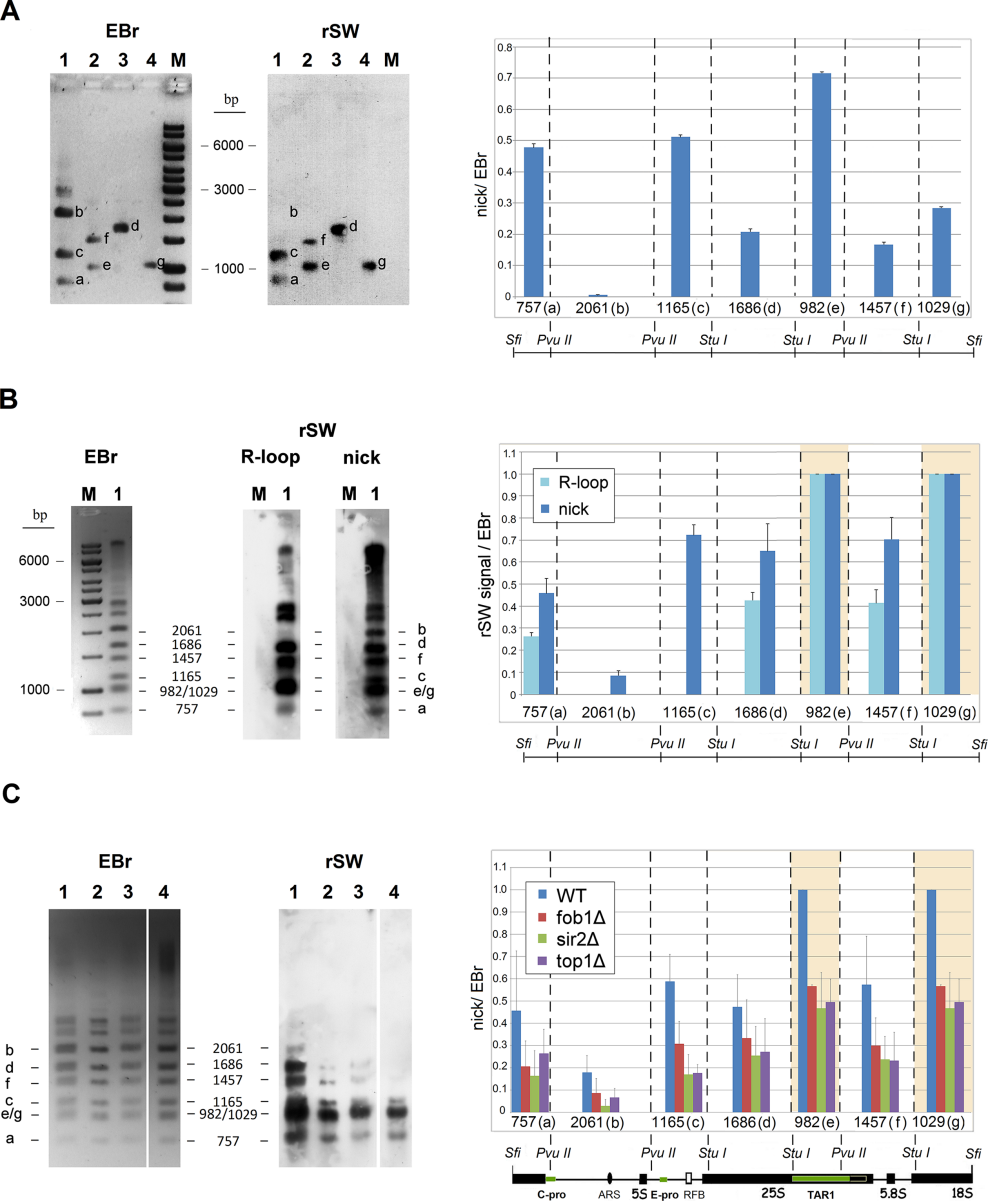
Nicks are unevenly distributed within the rDNA units, do not coincide with R-loops at the intergenic spacers and their incidence correlates well with rDNA silencing. (**A**) Reverse Southwestern blot (rSW) analyses of free 3′OHs in *S. cerevisia*e (WDHY199) rDNA units (see also Supplementary Figure S15). Agarose embedded, deproteinized gDNA was nick-translated in limiting conditions, digested with Sfi I and the fragments were separated by gel electrophoresis. The 9.1 kb rDNA units were cut out from the agarose gels and further digested with Stu I. After the next round of gel electrophoresis, the Stu I fragments (lane 1: 3983 bp; lane 2: 2419 bp; lane 3: 1686 bp; lane 4: 1029 bp) were cut out and further digested with Pvu II. M: 1 kb marker. Left sub-panel: EBr stained gel. Right: rSW anti-biotin staining of the blot. a-g: Pvu II generated bands of Stu I fragments. Histogram: fragment size (bp) and map position of the fragments are represented on the X-axis and on the map below, respectively, and labeling intensities (nick/EBr ratio: Biotin signal intensities of the fragments normalized to their corresponding EBr signal intensities) are shown on the Y-axis. (**B**) rSW analyses of free 3′ OHs and R-loops in *S. cerevisiae* (BY4741) rDNA units. Agarose-embedded and deproteinized gDNA samples were analyzed as in A. Immunostaining of blot was performed sequentially with the S9.6 antibody (sub-panel rSW, R-loop), then with an anti-biotin antibody (sub-panel rSW, nick). Lane 1, wild-type cells; M: 1 kb marker. EBr: EBr-stained gel. a–g: Pvu II and Stu I double-digest fragments of the rDNA units. For the description of X- and Y-axis see (A). S9.6 and biotin signal intensities of the fragments were each normalized relative to their corresponding EBr signal intensities. The band with the highest biotin/EBr or S9.6/EBr signal ratio on the gel was set to 1 on the relative scale. Note that the 982 bp and 1029 bp fragments migrated very closely together on the gel, so they were labeled as e/g and their cumulative intensities (marked by shaded areas) were determined. (**C**) rSW analyses of free 3′OHs in the rDNA of the *S. cerevisiae* WT (BY4741) and isogenic mutants *fob1*Δ, *top1*Δ, and *sir2*Δ. The agarose embedded, deproteinized DNA samples were analysed as in A. The size and position of restriction fragments (a–g) are labeled. Lane number, corresponding coloured bars in the histogram, and corresponding strain: 1, blue bars, WT; 2, red bars, *fob1*Δ; 3, green bars, *sir2*Δ; 4, purple bars, *top1*Δ. All the lanes shown are from the same gel. The X- and Y-axis are as in (A). For description of fragments e/g see (B). Bar charts in (A)–(C) represent mean values ± SEM obtained from three independent experiments. Positions of the fragments in (A)–(C) are mapped over the SfiI-rDNA unit (for a more precise mapping of fragment positions see Figure 6).

In order to enhance sensitivity and also to distinguish the nicks revealed by S1 digestion or urea/heat-denaturation from the constitutive, Fob1-dependent nicks carrying Top1- blocked 3′ termini at the RFB (17), we mapped endogenous ss nicks in the rDNA units by incorporating biotinylated nucleotides into free 3′OHs of gel-embedded samples by nick-translation followed by rSW blotting ((58); see flow-chart in Supplementary Figure S15). The localization of the nicks was made possible by using limiting nick-translation conditions (see the optimization in Supplementary Figure S6). Note also that the method has a wide dynamic range and is highly sensitive (see Supplementary Figure S16). The distribution of nicks with free 3′OH ends along the rDNA units was not uniform (Figure 5A). Remarkably, biotin-dUTP incorporation was barely detected in the fragments carrying the ARS sequences of rDNA. This observation is consistent with the results of the Southern hybridization experiments (Figure 4) and excludes rDNA replication firing from the primary causes of nick formation. Moreover, we noticed that three of the rDNA fragments that showed the highest incidence of nicks (Figure 5A, fragments ‘a’: 757 bp, ‘c’: 1165 bp, ‘e’: 982 bp) contain RNAP II promoter sequences (i.e. *C-PRO, E-PRO* and the promoter of *TAR1* gene, respectively (79–83)). Detection of R-loops with S9.6 staining on the same blots (Figure 5B) revealed strong coincidence with the nicks in several segments of the rDNA units. However, R-loops were completely absent from fragments ‘b’ (2061 bp) and ‘c’ (1165 bp), in line with (78). Thus, the high level of nick labeling in the 1165 bp fragment cannot be attributed to free 3′OHs of RNA origin, i.e. the elongation of R-loops by the DNA polymerase used for nick-translation could not contribute to the nick signals. This conclusion is in line with the results of the combing experiments shown in Supplementary Figure S1.

Finally, to further investigate if nick formation could be modulated by mutations affecting transcription, we compared the incidence of nicks along the rDNA units in strains with constitutively defective rDNA silencing (mutants *top1Δ, sir2Δ* and*fob1Δ*). Importantly, malfunctioning of rDNA silencing (an rDNA specific phenomenon central to the cooperation of RNAP II and I (83,84)) led to a decrease of nicking in the rDNA units in all of the three mutant strains (Figure 5C).

## DISCUSSION

Here we report that endogenous, non-random single-strand discontinuities/nicks are scattered along the *S. cerevisiae* genome. The nicks are distributed at ≤ 200 kb distance apart from each other on each strand, so as to yield ∼70–100 kb ds fragments upon further breakage at these predilection points. These findings extend our earlier reports on loop-size DNA fragmentation (25,36,37,85) as: (i) The nick-character of the breaks was corroborated by their independent distribution on the two DNA strands and in molecular combing experiments. (ii) By excluding superhelical tension as a factor eliciting the breaks, what further confirmed their endogenous nature. (iii) By determining the distribution of the nicks along both the whole gDNA and the rDNA in WT and *S. cerevisiae* mutants. The loop-size periodicity was recapitulated in the rDNA cluster, implicating sequence-unrelated factors in their generation/maintenance. The coincidence of the nicks with strategic sites of transcription regulation along the whole genome, including the rDNA cluster, is in line with the hypothesis that these nicks may be related to the promoter-proximal breaks of mammalian models (reviewed in ref. (39)). The analogy with the latter observations is supported by the association of nick incidence with active promoters, which is contrasted with the lack of nick accumulation in ARS regions. The demonstrated potential of gDNA for ds breakage at predilection points defined by nicks raises questions concerning the generally assumed ds character of the breaks detected in mammalian cells. The co-occurrence of nicks and R-loops in a fraction of gDNA and in certain regions but not in others of the rDNA units is pertinent to the role of these structures in genomic instability.

### Evidence for the endogenous character of the nicks

**Figure 6.**
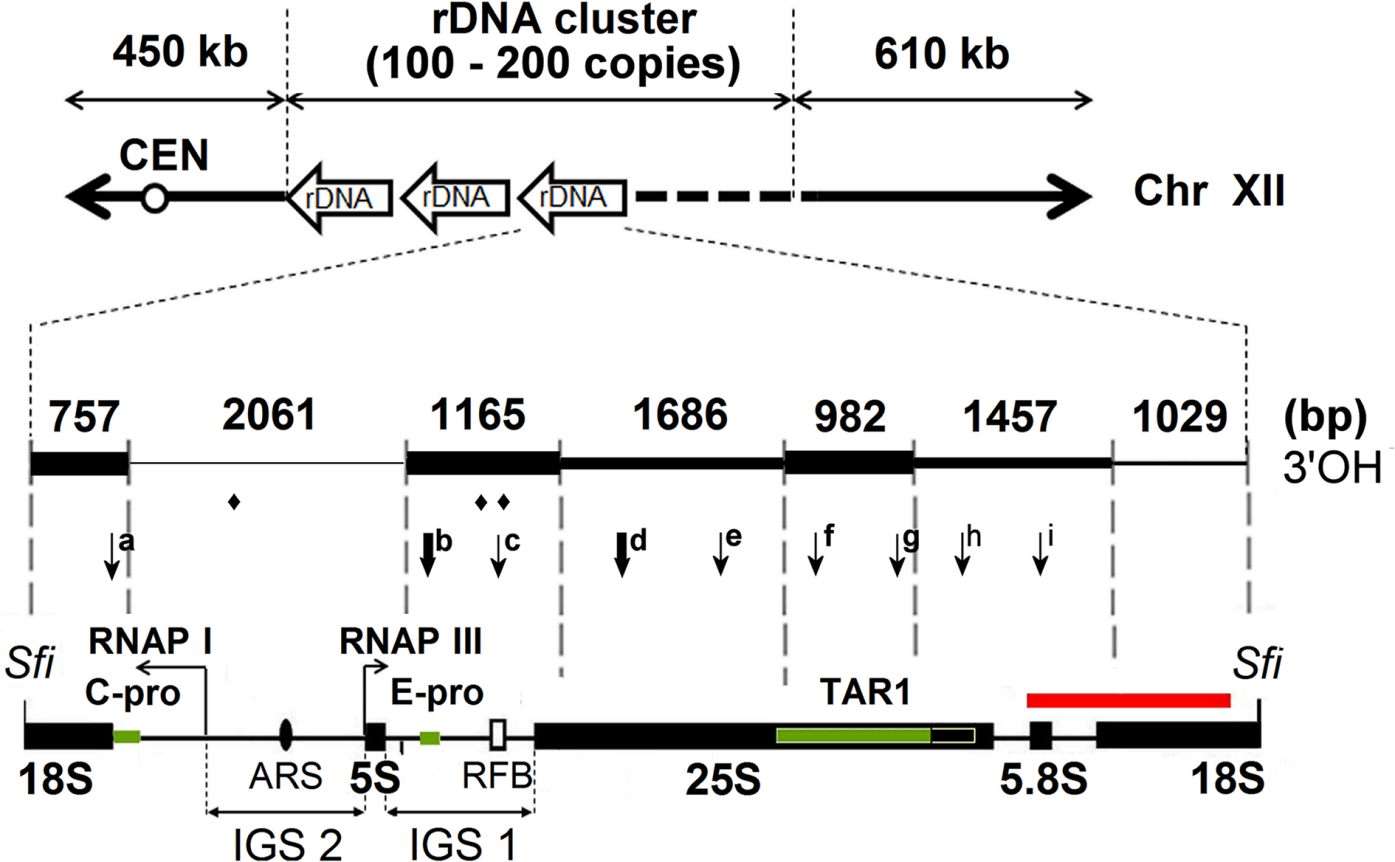
Overview of endogenous strand-breaks mapped within the rDNA unit. Black boxes indicate the 25S, 18S, 5.8S and 5S exons of the 9.1 kb rDNA unit. Black arrows show transcription from the RNAP I and III promoters. ARS and RFB are illustrated by a black oval and a white rectangle, respectively. CEN: centromere. Sfi: SfiI cleavage sites. The green bars show RNAP II promoters. Horizontal arrows show the direction of transcription from the RNAP I and III promoters. Arrows directed downward show the ss breaks detected in this work. Thick and thin arrows indicate the positions of strongly and weakly discernible breaks, respectively. The intervals of different thickness above the arrows indicate the 3′OH labeling intensity observed within the different restriction fragments in the rSW experiments (see Figure 5). Breaks labeled with rhombus (♦) were reported in (17,104,135). The position of the (strand-specific) Southern probe is indicated by a red horizontal bar. The numbers above the map show the size of the corresponding restriction fragments.

Several possible mechanisms for the artefactual generation of strand discontinuities in our experimental circumstances have been excluded in earlier publications (25,26,33,36,85), and the role of superhelical tension has been ruled out in this paper (Supplementary Figure S5). Our microarray experiments that were conducted using fixed cells also support this notion. Further strong arguments are provided by the non-random localization of nicks found by several methods in this study. Mapping of the nicks within the rDNA units by Southern blot analyses of denatured chr XII DNA samples revealed several strand-specific breaks, at discrete sites. On the other hand, the IGS2-ARS-containing 2 kb region was poor in breaks (Figures 4-6). The relative lack of breaks in the ARS regions (as shown in gDNA by microarray experiments (Figure 2), and within the rDNA cluster by Southern hybridization and rSW (Figures 4 and 5, respectively) provides compelling evidence that some genomic regions are spared of nicking. rSW is a highly sensitive method that detects nicks carrying 3′OH in a particular region, so all the breaks within the restriction fragment are detected regardless of their exact position. Since this method was applied to detect nicks in a naturally amplified locus, the nearly complete absence of nicks from IGS2 in some experiments (e.g. Figure 5A) suggests that the level of random nicking in our setup is usually very low. The facts that the epigenetic state of both IGS1 and IGS2 is heterochromatic (86,87) and IGS1 harbors much more nicks than IGS2, argue that the site-specific nicks reported here cannot be attributed to nonspecific nucleases acting in open chromatin regions. The low level of nicks in the rARS region in spite of the fact that one fifth of the rDNA units are engaged in replication during a single cell cycle also suggests that accessibility is not the decisive factor in the generation of the nicks. The fact that the incidence of nicks is linked to transcriptional processes (see Figures 2E, 5C and Supplementary Figure S9) supports the notion that the nicks are of physiological function and origin. The *S. pombe* genome, distant from that of the bakers’ yeast in evolution, also harbours endogenous nicks at loop-size intervals, in line with our hypothesis that the promoter-proximal, gene activity-dependent DNA breaks observed in mammalian cells and those described herein are related.

Collectively, our data suggest that the nicks detected in our assays exist before cell lysis, i.e. they are present in the gDNA, including the rDNA, of live cells, and become manifest as ds breaks only after lysis under conditions that allow unfolding and diffusive motility of chromosomal DNA.

### The incidence of nicks is connected with transcriptional processes

Accumulation of the nick signal at RNAP II promoters and the finding that active gDNA promoters preferentially harbor nicks (Figures 2, 6 and Supplementary Figure S7A) imply that the ss breaks appear in the context of a RNAP II-related transcriptional activity. According to Supplementary Figure S9, they are associated with initiation rather than elongation.

In the rDNA cluster, some of the DNA breaks that mapped at the locus coincide with the RNAP II promoters embedded in the rDNA units (see Figure 6). The finding that nicks with free 3′OH ends co-localize with RNAP II promoters also within the rDNA is surprising, because nucleoplasmic gDNA gene transcription and the RNAP II related nucleolar transcriptional processes are fundamentally different. Whereas the RNAP II controlled transcriptional processes at bulk gDNA lead mainly to the synthesis and concomitant processing of protein coding primary mRNA transcripts, RNAP II appears to transcribe mostly noncoding RNA in the nucleolus (except for *TAR1*; see below). The RNAP II promoters in the rDNA are *E-PRO, C-PRO* and *TAR1* (see Figure 6). *E-PRO*, a bidirectional promoter, resides in the rDNA units close to the RFB and has a role in the regulation of rDNA amplification via its noncoding RNA products (54,79). Another bidirectional RNAP II promoter, *C-PRO* (a member of the Cryptic Unstable Transcripts; CUTs), lies in the IGS2 region and its biological role is presently unknown. CUTs represent 200–500 b noncoding RNAs that are rapidly degraded in an Nrd1-dependent pathway and are the major products of pervasive transcription (88–90). *TAR1* (Transcript Antisense to Ribosomal RNA), is embedded into the 25S exon on the antisense strand, is expressed at very low levels and encodes a protein which is localized in the inner membrane of mitochondria (80,91). The levels of transcripts originating from the IGS promoters is low in WT cells, due to rDNA silencing. In this process, the highly active transcriptional activity of RNAP I at the 35 S rDNA leads to the suppression of RNAP II activity at the IGSs (83,84,92–95). Silencing appears to be the direct consequence of histone deacetylation by Sir2. The Sir2 enzyme-containing protein complex is recruited to Fob1 (in a Top1-dependent manner (17,84)) and also to the Pol I promoter (96).

In view of the fundamental differences between RNAP II functions in rDNA and in the rest of the genome, the role and regulation of nick generation may be very different in the two locations. Indeed, opposite to their prevalence at active promoters in the bulk of gDNA, the nicks accumulate at the sluggish RNAP II promoters in rDNA and avoid the much more active RNAP I and RNAP III promoters. The strikingly similar behaviour of three mutants (*top1Δ, sir2Δ, fob1Δ*) affecting the same pathway of regulation of rDNA silencing (84,96,97) strongly argues for a scenario where nicks play a role in transcriptional regulation also in the rDNA. However, the molecular interpretation of the links between nick incidence and transcriptional regulation awaits a better understanding of the intertwined and still mysterious regulation of RNAP I and RNAP II activities at the rDNA locus (82,90,93,94), involving the antagonistic activity of RNAP I and II (94) on the one hand, and the contribution of RNAP I transcription to the maintenance of RNAP II silencing (96) on the other.

Thus the nicks detected in our assays are associated with the transcriptional regulation involving RNAP II also at the rDNA locus. On the other hand, the nicks appear not to be related to DNA replication, since they occur in G1- and G2/M synchronized cells alike (see Supplementary Figure S14) and do not accumulate in the ARS-containing regions (see Figures 2, 4, 5 and 6).

### Possible molecular mechanisms of nick generation

Detection of breaks having free 3′OH end formally rules out the role of Top1 in their generation, since this enzymatic reaction would yield covalent enzyme-DNA complexes, i.e. Top1ccs (98,99).

In yeast, Top1 and, often interchangeably, Top2 play important roles both in initiation and elongation of transcription (100–103). In addition to its general role in resolving superhelical stress during transcription that would give rise to random nicks, Top1 is responsible for the generation of two site- and strand-specific, persistent nicks (coinciding with ‘c’ in Figure 6) at the rRFB (17,104). The presence of these known, Top1- and further, possibly Top3-related nicks that are also localised at the RFB (17,104) (see ♦, ♦♦ in Figure 6), may be mainly responsible for the observation that few combed chr XII molecules were detected carrying label on both ends, as opposed to combed gDNA (see Supplementary Table S5). The nicks detected via their free 3′OH in the rSW experiments must be Top1-unrelated. Indeed, analysis of the three rDNA silencing deficient strains revealed nicks at the same localization, albeit at reduced incidence (see Figure 5C). In view of the 5′-tyrosine linkage in the cleavage complex formed by these enzymes, it is much more likely that Top2 (or perhaps Top3) is responsible for the generation and maintenance of the breaks at the rDNA (detected both by Southern hybridizations and the rSW procedure) in sites ‘a-b’ and ‘d-i’ of Figure 6. We note, however, that the involvement of Top1 cannot be ruled out unambiguously, since this enzyme may be released from the Top1cc state by e.g. Tdp1 (Tyrosyl-DNA Phosphodiesterase 1; (105)) which would generate free 3′OHs available for labeling. Free 3′OH ends can also be generated in the course of base excision repair. However, neither the preferential generation of such lesions during RNAP II elongation (106), nor their faster repair at nucleosome depleted regions (107) correlate with our observations. On the other hand, in normal mammalian cells and conditions without exposure to mutagens or any other forms of stress, repair factors appear to function at the promoter-proximal, Top2-induced transient lesions that accompany transcriptional activation (39,108). A scenario where the DNA breaks observed herein could be generated by Top2 would be highly attractive: Top2ccs with a single nicked DNA strand (such as those mapped in human colon cancer cells (9)) could tether the bases of loops in concert with topological relaxation with its concomitant nucleosome destabilizing effect (67). Addressing the possible role of Top2 (or Top3) directly would not be trivial since these enzymes are essential for growth in budding yeast (109,110), have multiple roles in transcriptional regulation (15), and any break made by Top2 before the transfer of a conditional mutant to restrictive conditions is expected to persist even after the enzymes are down-regulated, inhibited, subjected to heat-reversal, or upon inactivation (111–114). The findings that in the bulk of gDNA Top2 contributes to the assembly of the RNAP II preinitiation complex (102,103), whereas the depletion of Top2 appears to unslilence RNAP II related ncRNA transcription in rDNA (77), fit the correlation between nick incidence and RNAP II promoter activity in gDNA on the one hand, and between nicking in rDNA and rDNA silencing on the other.

### The nicks may be transient

The ∼70–100 kb average size of the ds fragments bordered by endogenous nicks was measured by different approaches: (i) electrophoretic sizing of large fragments (also by CHEF, to avoid the focusing artefact of FIGE (115)), and (ii) by microscopic observation of the DNA fragments generated via stretching-induced breaking (116) at nicks in the molecular combing experiments. On the other hand, the distribution of 3′OH-labeled nicks mapped along the gDNA on microarrays coincided with the promoters of active protein coding genes in general. Since these comprise the majority of yeast genes (117), the TSSs potentially harboring nicks may be ∼2 kb apart from each-other, assuming that sampling was random. It follows that any particular endogenous nick detected in the microarray experiments along the genome must be present only in a minor subpopulation of cells at the time of fixation and nick-labeling, in line with Supplementary Figure S8D. The size of this subpopulation is determined by the sensitivity of nick detection and by biological reasons related to the mechanism of nick generation.

The promoter-proximal association of Top2 has been documented in (102,103,118). If Top2 was responsible for the generation of the nicks observed in our assays, stochasticity could arise in two ways: (i) the active promoters transiently interact with a limited pool of enzymes in a competitive manner, or (ii) each active promoter is permanently Top2-associated, but the enzyme at any particular promoter spends a small part of its catalytic cycle in the Top2cc state, when the relative duration of this time window would determine the average distance of nicks present in the DNA at any given time point. In summary, we propose that the generation of nicks may be a random process preferentially involving active RNAP II promoters.

### Higher-order organization of the chromatin and the distribution of nicks

The generation of nicks occurs only in every ∼10th unit of the rDNA on the average (Figure 3B and C), excluding sequence related factors from the primary determinants of nick generation on the one hand, and demanding topological interpretation on the other. Chromatin loops ranging from 20–300 kb have been described in *S. cerevisiae* (119–126). The differences in the size estimates may be due to the differences of the experimental systems, the varied experimental criteria of loop definition and the dynamic features of chromatin determining the loop subpopulations sampled by the particular method. The distribution of nicks, as revealed by independent methods, is in the middle of this wide range. Hence we asked if higher-order organizational features of the chromatin, a question of special interest in the case of the repetitive rDNA locus, would coincide with nick distribution. In view of the obvious demand for superhelical stress dissipation and means of disentanglement in the case of looped domains (127), their anchorage, be it a transient event, e.g. involving topoisomerases, would stand to reason.

Only ∼50% fraction of the 100–150 rDNA units is transcriptionally active (83,95), with extensive variations depending on the actual metabolic conditions of the cells (86). The loop-size defined by the constitutive Top1ccs involved in rDNA silencing (17) has not yet been reported. The silenced units are thought to be packaged into regular nucleosomes while the transcriptionally inactive IGS1 and IGS2 regions of active rDNA units exhibit complex features (86). Since the distribution of silenced and active units appears to be random (95), the periodicity of nick accumulation in every 10th unit, based on independent assays (see Figure 3 and Supplementary Table S3), cannot be readily accounted for by the 1:1 dichotomy of active and silenced units. The incidence of active replication origins (1:5) more closely corresponds to the average incidence of nicks, even without considering ARS clustering (48). However, in view of the lack of accumulation of nicks in the ARS regions and their presence at the promoters also in G1 cells (data not shown), this imperfect correlation may not be meaningful. Known manifestations of the higher-order organization of the locus include IGS1-IGS2 looping via interaction between RFB and ARS involving RNAP II (128), and the formation of mutually exclusive promoter-terminator loops by the rDNA genes controlled by the different RNA polymerases (49). Whether these interactions occur within a single unit or involve inter-repeat looping, as pointed out in (127), is an open question.

Thus, the incidence of nicks detected in the rDNA cluster does not seem to exactly coincide with any of the periodicities observed so far. Determination of these relationships will perhaps be made possible by superresolution or electronmicroscopic localization of nicks relative to the other known structural and functional elements of the rDNA units.

### Implications for genomic instability

Our electrophoretic and molecular combing data both suggest that endogenous nicks are present on the two strands of gDNA in a non-apposed manner, i.e. in an arrangement what is expected to maintain ds chromosomal continuity when the DNA is kept and handled within agarose plugs. By analogy to observations made in the combing experiments that involve considerable stretching of the DNA molecules (116), endogenous ss breaks likely serve as predilection points for the loop-size ds fragmentation of the gDNA observed upon cell lysis in the absence of embedding, due to shearing forces (e.g. see Supplementary Figure S2A, lane 8). This interpretation is in line with early observations made on shear-induced ds breakages of nicked T5 phage DNA molecules (129). Thus, detection of DSBs in experimental conditions devoid of embedding may in fact reveal endogenous ss breaks.

The mechanical vulnerability of the DNA at nicks demonstrated herein may reflect a similar susceptibility to mechanical breakage in living cells. Furthermore, demonstration of the endogenous character of these breaks entails the presence of highly recombinogenic free ends (including free 3′OH termini) *in vivo*. In view of the possibility that the breaks are caused by Top2, it is worth mentioning that in mammalian cells, promoter proximal Top2β, in addition to being implicated in transcriptional regulation (39), has been incriminated as a crucial player in chromosomal translocations leading to cancer (39,130–132). A further intriguing connection with our findings is represented by recent observations implicating Top2 in the aging of *S. cerevisiae* (109).

Co-localization of nicks and R-loops could be observed both in the bulk of gDNA and in rDNA. According to our rSW experiments, 7 kb out of the 9.1 kb rDNA unit harbored both entities. The fact that the IGS1 and IGS2 regions are devoid of R-loops is in line with the transcriptional silencing of these regions and correlate with earlier S9.6-ChIP-seq data (78). In agreement with the rSW results, R-loops were frequently detected at the combed chr XII ends (see Figure 3H), similarly to the endogenous nicks. In view of the fact that R-loops contain a ss region (demonstrated by their SSBP staining, see Figure 3H), these structures may be also prone to mechanical breakage upon combing. These are potentially important observations in view of the genomic instability accompanying R-loop misregulation (21,22,70,133).

## DATA AVAILABILITY

Microarray datasets generated and analyzed in this study have been submitted to Gene Expression Omnibus (GEO), with the following accession number: GSE110218.

## Supplementary Material

gky743_supplemental_files
