# Supplementary material for: Endogenous single-strand DNA breaks at RNA polymerase II promoters in Saccharomyces cerevisiae
Source: Nucleic Acids Res. 2018 Aug 24;46(20):10649–68. doi: 10.1093/nar/gky743 (PMC6237785; doi:10.1093/nar/gky743)
Supplement: gky743_supplemental_files [file gky743_supplemental_files.zip › gky743_supplemental_files.pdf]

Supplementary Data for

Endogenous single-strand DNA breaks at RNA polymerase II promoters in *Saccharomyces cerevisiae*

Éva Hegedüs[1], Endre Kókai[2], Péter Nánási[1], László Imre[1], László Halász[3], Rozenn Jossé[4], Zsuzsa Antunovics[5], Martin R. Webb[6], Aziz El Hage[7], Yves Pommier[4], Lóránt Székvölgyi[1,3], Viktor Dombrádi[2] and Gábor Szabó[1]

[1]Department of Biophysics and Cell Biology, Faculty of Medicine, University of Debrecen, Hungary

[2]Department of Medical Chemistry, Faculty of Medicine, University of Debrecen, Debrecen, Hungary

[3]MTA-DE Momentum Genome Architecture and Recombination Research Group, Department of Biochemistry and Molecular Biology, Faculty of Medicine, University of Debrecen, Debrecen, Hungary

[4]Developmental Therapeutics Branch and Laboratory of Molecular Pharmacology, Center for Cancer Research, National Cancer Institute (CCR-NCI), NIH, Bethesda, MD, USA

[5]Department of Genetics and Applied Microbiology, Faculty of Science and Technology, University of Debrecen, Debrecen, Hungary

[6]The Francis Crick Institute, London NW1 1AT, United Kingdom

[7]Wellcome Trust Centre for Cell Biology, University of Edinburgh, Edinburgh, United Kingdom.

Correspondence: szabog@med.unideb.hu

<u>**Contents**</u>**:**

Supplementary Materials and Methods

1. Cultivation of *S. cerevisiae* and *S. pombe*
2. Synchronization of *S. cerevisae* cells in G1 and G2/M phases
3. Ethidium bromide treatment of spheroplasts
4. Preparation of agarose plugs containing *S. pombe* chromosomes
5. Extraction of gDNA from agarose plugs and from cell suspensions
6. Gel electrophoretic techniques
7. Preparation of biotin-labeled PCR products
8. Optimization of dNTP/ddNTP ratio for limited nick translation using nickase digested PCR products
9. Global chip-on-beads assay

Supplementary Tables

- Table S1: Frequency of labeled fibers among combed DNA molecules
- Table S2: Frequency of fiber termini with nick or R-loop labeling
- Table S3: Nick density measured in combed gDNA and chr XII DNA of non-synchronized and G1 synchronized cells
- Table S4: Fiber lengths statistics of nick-labeled gDNA and of λ DNA
- Table S5: Percentage of end-labeled and internally labeled fibers

Supplementary Figures

- Figure S1: Detection of R-loops and nicks on combed gDNA from non-synchronized *S. cerevisiae* cells, without or with RNase treatment.

- Figure S2: Gel electrophoretic analysis of *S. cerevisiae* gDNA demonstrates nicks arranged on the two DNA strands in a non-apposed manner
- Figure S3: S1 nuclease sensitive sites at loop-size intervals in the *S. pombe* genome.
- Figure S4: Work-flow of urea/heat-denaturing gel electrophoresis
- Figure S5: Effect of the superhelical state of gDNA on the incidence of ss discontinuities
- Figure S6: Optimization of dNTP/ddNTP ratio for nick translation in limiting conditions
- Figure S7: Nick incidence correlates with gene expression at TSSs and is not related to TTSs.
- Figure S8: Nick ChIP-chip analyses of endogenous nicks and exogenous nicks.
- Figure S9: Global chip-on-beads analyses of nick – RNAP II colocalization.
- Figure S10: Scheme of two-dimensional gel electrophoretic analyses
- Figure S11: Two-dimensional gel electrophoretic analyses of nicks in *S. pombe* chromosomes
- Figure S12: Mapping of DNA breaks within the rDNA locus using single-stranded rDNA probes.
- Figure S13: Dependence of the apparent fragment size on the order of treatments during detection of nicks in the rDNA units
- Figure S14: Mapping of DNA breaks within the rDNA locus in G1- and G2/M-synchronized cells
- Figure S15: rSW blot procedure for detection of nicks in rDNA units
- Figure S16: Calibration of rSW procedure

Supplementary References

Supplementary Materials and Methods

Cultivation of S. cerevisiae and *S. pombe*

The *Saccharomyces cerevisiae* strains used in this study (Table 1) were grown as previously described [1]. Briefly, yeast cells from a single colony were inoculated and grown under non-selective conditions in liquid YPD medium (1% yeast extract, 2% bactopepton, 2% glucose, pH 5) at 30°C, to a concentration of $\sim 2\times10^7$ cells/ml (logarithmic phase, OD_{600}=1). The viability of cells from log phase was found ~100 % by colony forming, i.e. normal, non-apoptotic yeast cells were used in our studies. The *Schizosaccharomyces pombe* L972h^-(wild-type) cells were grown in liquid YEL (0.5% yeast extract, 3% glucose, pH 5) at 30°C to a concentration of $\sim 5\times10^6$ cells/ml (in log phase).

Synchronization of S. cerevisiae cells in G1 and *G2/M phases*

- *G1 phase:* The *S. cerevisiae bar1Δ* mutant cells (BY4741 background) were grown to OD_{600}=0.7, then cells were incubated in the presence of α-factor (50 ng/ml final concentration, Sigma) for 1.5 hours at 30°C. The synchronization was controlled under the microscope.
- *G2/M phase:* BY4741 cells were grown to OD_{600} = 1, then incubated for 1.5 hours in the presence of nocodazole (20 μg/ml final concentration; Sigma). The synchronization was controlled by flow cytometry.

Ethidium bromide treatment of spheroplasts

Preparation, S1 digestion and urea/heat-treatment of the agarose-embedded *S. cerevisiae* gDNA were carried out as previously described [1], but all solutions were supplemented with ethidium bromide (EBr) to change the superhelical state of gDNA embedded into agarose plugs, at a final concentration of 0, 0.5, 1, 2.5, 5, 10, 25, 50 μg/ml. All procedures were performed in the dark. The EBr entered the intact *S. cerevisiae* cells within a few minutes, as detected by fluorescence microscopy.

Preparation of agarose plugs containing S. pombe chromosomes

S. pombe cells were harvested and washed twice in 50 mM EDTA (pH 8.0), then resuspended in CPES buffer (25 mM citric acid, 0.12 M Na_2HPO_4, 20 mM EDTA, 1.2 M sorbitol) containing 15 mg/ml lysing enzyme (Sigma). The spheroplasting process was

controlled under the microscope using the SDS test. Samples were mixed with an equal volume of 1.5 % LMP agarose dissolved in 0.9 M sorbitol/0.125 M EDTA. Aliquots were allowed to harden in sample molds at 4°C for 5 minutes. Each plug contained ~1.5×10^7 cells. The plugs containing yeast spheroplasts were digested with 0.5 mg/ml Proteinase K (Thermo Fisher Scientific) in lysing solution (0.5 M EDTA, 10 mM Tris-HCl, 1% SDS, pH 8.0) at 55°C for 2 days, then washed with TE and treated by 0.75 μM PMSF at 37°C for 10 minutes in order to inactivate residual proteinase activity. Finally, the plugs were washed with TE and stored in the same buffer at 4°C.

Extraction of gDNA from agarose plugs and from cell suspensions

Agarose plugs containing deproteinized *S. cerevisiae* spheroplasts were soaked in 1×TAE (40 mM Tris-acetate, 1 mM EDTA, pH 8.0) and incubated at 65°C to melt the agarose and digested with β-agarase enzyme (Promega) at 42°C for 16 hours according to the manufacturer's instructions. DNA was purified by phenol-chloroform extraction and isopropanol precipitation as described below.

DNA preparation from *S. cerevisiae* cell suspensions was carried out as previously described [1, 2]. Briefly, *S. cerevisiae* cell cultures were fixed by adding ethanol to 50% and EDTA to 25 mM final concentration and incubation at −20°C overnight. The fixed cells were washed with TE and incubated in digestion buffer (1 M sorbitol, 10 mM Na_2HPO_4 (pH 7.2), 10 mM EDTA, 1% β-mercaptoethanol and 2 mg/ml lyticase enzyme (Sigma-Aldrich)) at 37°C for 30 minutes. The resulting spheroplasts were harvested and incubated in lysis buffer (200 μg/ml Proteinase K, 0.5% SDS, and 50 mM EDTA, pH 8.0) at 54°C for 30 minutes. Then 0.4 volume of 5 M K-acetate was added and the sample was placed on ice for 20 minutes then centrifuged at 12,000 *g* for 30 minutes at 4°C. The nucleic acids in the supernatant were precipitated with an equal volume of isopropanol. The pellet was incubated with 0.2 mg/ml RNase A (Thermo Fisher Scientific) in TE at 37°C for 30 minutes. DNA was precipitated with 0.5 volume of 7.5 M ammonium acetate and 1.5 volume of isopropanol. The pellet was rinsed with 70% ethanol, dried and dissolved in TE.

Gel electrophoretic techniques

❖ *Conventional gel electrophoresis:* Non-denaturing and urea/heat-denaturing gel electrophoresis were performed in 1.2% agarose (from Seakem) gel in 1×TAE for 15 hours at 45 V in the cold room. For urea/heat-denaturing, both the agarose gel (urea-agarose gel) and

running buffer was supplemented with 1 M urea. The agarose plugs were preincubated with 8 M urea/TE for 40 minutes at room temperature, then heat-denatured for 5 minutes at 80°C.

❖ *Field inversion gel electrophoresis (FIGE):* Non-denaturing and urea/heat-denaturing FIGE were carried out as previously described [1], using an MJ Research PPI 200 Power Inverter apparatus. The gels were run at 6 V/cm in the cold room, using running parameters set to maximize separation in 50 – 400 kb range. For urea/heat-denaturing, the agarose plugs were preincubated with 8 M urea/TE for 40 minutes at room temperature, then heat-denatured for 5 minutes at 80°C. In the case of alkaline denaturation, the plugs were soaked in alkaline solution (50 mM NaOH, 1 mM EDTA) for 20 minutes at room temperature and then the DNA fragments were run in 1 % urea-agarose gels (containing 1 M urea) in 1×TAE containing 1M urea. After gel electrophoresis, the gels were stained with 0.5 μg/ml ethidium bromide (EBr) for 30 minutes and destained in water for 1 to 2 h.

❖ *Contour-clamped homogeneous electric field electrophoresis (CHEF):* 1% agarose gels were prepared in 0.5 × TBE (45 mM Tris-borate, 1 mM EDTA, pH 8.3). Separation of chromosomal DNAs of *S. cerevisiae* was carried out using CHEF Mapper XA Pulse Field Electrophoresis System (Bio-Rad Laboratories, Inc., Hercules, California, USA) with 60 s initial and 90 s final switch time for 24 hours at 6 V/cm at 14 °C with an included angle of 120° in 0.5× TBE buffer. To resolve loop-size fragments it was programmed for a switch time of 1s to 25 s for 24 hours at 6 V/cm at 14 °C with an angle of 120°. After gel electrophoresis, the gels were stained with EBr and destained in water, as described above. Separation of chromosomal DNAs of *S. pombe* was carried out in 1% agarose gel prepared in 0.5×TBE using CHEF Mapper XA Pulse Field Electrophoresis System (Bio-Rad Laboratories) with 5.400 s initial and 5.400 s final switch time for 70 hours and then 100 s initial and 4.700 s final switch time for 50 hours at 1.2 V/cm at 14 °C with an included angle of 120° in 0.5× TBE buffer.

❖ *Two-dimensional gel electrophoresis:* Chromosomal DNA molecules of *S. cerevisiae* and *S. pombe* were separated from each other in 1 % agarose gels (prepared in 0.5×TBE) by CHEF Mapper XA PFGE system (Bio-Rad Laboratories, Inc.) in the first dimension. A whole lane containing the separated chromosomes was cut out, S1 treated and run in a perpendicular dimension by FIGE, or the bands containing the individual chromosomes were cut out, subjected to urea/heat-denaturation and analyzed by FIGE on urea-gels. This work-flow is similar to what was reported in [3, 4].

Preparation of biotin-labeled PCR products

PCR was performed in 50 µl of 1×reaction buffer (10 mM Tris-HCl, 50 mM KCl, 0.08 % Nonidet P-40, pH 8.8) supplemented with 3 mM $MgCl_2$, containing 280 ng template DNA (*S. cerevisiae* gDNA), 20 pmol of both primers (sense: 5'-ATGATTAGAGGTTT-3', IDT; antisense: 5'-biotin-TCCGGGGTGATAAGCTGTTA-3', IDT), the triphosphates (dATP, dTTP, dGTP and dCTP; Promega Life Sciences) at 0.25 mM concentration, and 2.5 U Taq polymerase (Thermo Fisher Scientific) per reaction, yielding 712 bp ds PCR products carrying biotin on the 5' end. The PCR reactions were performed in an Hybaid thermal cycler and run with a temperature profile of 2 minutes at 94°C, 40 seconds at 60°C and 40 seconds at 72°C followed by 35 cycles of 80 seconds at 94°C, 40 seconds at 60°C and 40 seconds at 72°C. The 35 cycles were followed by 10 minutes at 72°C.

Optimization of dNTP/ddNTP ratio for limited nick translation using nickase digested PCR products

Long PCR reaction was performed in 50 µl of 1×long PCR buffer supplemented with 1.5 mM $MgCl_2$, containing 300 ng *S. cerevisiae* gDNA (prepared as described in Materials and Methods), 20 pmol of the rDNA primers (sense: 5'-GGG GAT CGA AGA TGA TCA GA-3', IDT; antisense: 5'- TCC GGG GTG ATA AGC TGT TA-3', IDT), the four triphosphates dATP, dTTP, dGTP and dCTP (Promega Life Science) at 0.25 mM concentration per reaction, and 1.25 U of the Long PCR Enzyme mix (Thermo Fisher Scientific). PCR reactions were performed in an Hybaid thermal cycler and run with a temperature profile of 5 minutes at 95°C, then 10 cycles of 20 seconds at 95°C, 30 seconds at 60°C and 3 minutes at 68°C, followed by 15 cycles of 20 seconds at 95°C, 30 seconds at 60°C and 7 minutes at 68°C. The cycles were followed by 10 minutes incubation at 68°C. The PCR products were purified by PCR quick-spin PCR Product Purification Kit (Intron).

9 µg of the 3282 bp PCR product was digested with 100 U/ml Nb.Bpu10I nickase enzyme, cutting at a single site (see inset), in 1× Buffer R (10 mM Tris-HCl (pH 8.5), 10 mM $MgCl_2$, 100 mM KCl, 0.1 mg/ml BSA), at 37°C, for 30 minutes, then supplemented with TE to 500 µl. Nb.Bpu10I digested PCR products were mixed with an equal volume of 1.5 % LMP agarose prepared in 1×DNA polymerase I buffer, placed into sample molds and allowed to solidify resulting in 900 ng PCR product /100 µl plug. Agarose plugs containing Nb.Bpu10I digested PCR products were nick translated using 150 U/ml DNA polymerase I (Thermo Fisher Scientific) in 1×DNA polymerase I buffer containing the dNTP mix (dTTP,

dATP, dCTP and dGTP) and the ddNTP mix (ddATP, ddTTP, ddCTP and ddGTP) applied at the concentrations indicated in the Figure legend, and incubated for 30 minutes on ice to equilibrate before 20 minutes of the polymerization reaction conducted at 37°C. The urea/heat-denatured samples were run in an 1.2 % urea-agarose gel.

Global chip-on-beads assay

❖ *Preparation of nuclei*: The pellet of 400 ml log phase culture of *S. cerevisae* cells was resuspended and fixed in 10 ml freshly prepared 4% paraformaldehyde dissolved in 1×PBS, rotating the cells for 10 minutes at room temperature. After quenching in 2.5 M glycine and washing twice with 50 mM Tris-HCl (pH 7.5) /30 mM DTT, the cells were treated with 2 mg/ml lyticase dissolved in 0.9 M sorbitol/0.125 M EDTA/10 mM DTT at 37 °C for 30 minutes. The spheroplasts were washed twice with 10 ml ice-cold 0.9 M sorbitol/0.125 M EDTA, lysed in 4 ml ice-cold buffer N (30 mM HEPES pH 7.6, 3 mM DTT, 25 mM Na_2SO_4, 5 mM $MgCl_2$, 1 mM EDTA, 10% glycerol, 0.5% NP-40, 7.2 mM spermin, 1 mM PMSF) and disintegrated using a Dounce homogenizer on ice for 60 min.

❖ *Nickase treatment and nick translation*: The nuclei were washed three times in ice cold 1×PBS/EDTA (1×PBS, 5 mM EDTA) followed by washing in nickase buffer (10 mM Tris-HCl pH 8.0, 50 mM NaCl, 10 mM $MgCl_2$, 1 mg/ml BSA). Random nicks were introduced into the DNA of control samples using the frequent cutter Nt.CviPII nickase enzyme (recognition site: CCD; New England Biolabs Inc., Ipswich, Massachusetts, USA) at a final enzyme concentration of 0.5 U/ml. Limited nick translation of the samples harboring only endogenous nicks and of the nickase treated control was performed as described in Materials and Methods.

❖ *Fragmentation of chromatin, microbead capture*: The nick-translated samples of nuclei were resuspended in 700 µl IP lysis buffer (50 mM HEPES-KOH pH 7.5, 140 mM NaCl, 1 mM EDTA, 1% Triton X-100, 0.1% Na-deoxycholate) and disintegrated using a FastPrep-24™ 5G bead beater (MP Biomedicals, Santa Anna, California, USA) at default setting for *S. cerevisae*, applying 200 µl glass beads/sample. Disintegration of the nuclei was checked under the microscope. After removal of the beads, the samples were sonicated with a Bioruptor Plus instrument (Diagenode, Denville, New Jersey, USA) for 3 cycles (1 cycle: 30 sec ON, 30 sec OFF, for 5 minutes) and spun down in an Eppendorf centrifuge at maximum speed at 4°C for 5 minutes. The supernatants were transferred into a clean Eppendorf tube and

the fragment size was determined by gel electrophoresis in a proteinase K treated aliquot (~500 bp in the experiment shown in supplementary Figure S9). The streptavidinated Dynabeads (Thermo Fisher Scientific, Waltham, Massachusetts, USA) were blocked with 1% BSA dissolved in 1×PBS/EDTA and washed three times with 1×PBS/EDTA. 60 000 beads were added to the sonicated chromatin and rotated at 4°C overnight. The beads were washed with 1×PBS/EDTA and embedded into agarose in an 8-well chamber (Ibidi, Martinsried, Germany) as described in [5].

❖ *Immunofluorescence labeling*: The agarose embedded Dynabeads were washed twice with ice cold lysis buffer, twice with lysis buffer supplemented with 360 mM NaCl, twice with ice cold washing buffer (10 mM Tris-HCl pH 8.0, 250 mM LiCl, 0.5% Na-deoxycholate, 1mM EDTA), finally twice with ice cold 1×PBS/EDTA. Indirect immunofluorescence labeling was performed using rabbit polyclonal anti-RNAP II Ser2-P (Abcam, Cambridge, UK; 1 mg/ml) or rabbit polyclonal anti-RNAP II Ser5-P (Abcam, Cambridge, UK; 1 mg/ml) diluted in 200 μl 1×PBS/EDTA/1% BSA at 4°C, overnight. The antibodies were applied to the wells at a titer of 1:400. After labeling with the primary antibodies, the beads were washed with ice cold 1×PBS/EDTA three times for 10 minutes. Labeling with the secondary antibody were performed in 200 μl 1×PBS/EDTA on ice for two hours, using Alexa fluor 488 (A488) conjugated goat anti-rabbit IgG (Thermo Fisher Scientific, Waltham, Massachusetts, USA; 2 mg/ml). The secondary antibody was used at a titer of 1:800, diluted in 1×PBS/EDTA. After labeling, the agarose embedded beads were washed with ice cold 1×PBS/EDTA three time and stained with 200 μl 12 μg/ml propidium–iodide (PI; in 1×PBS/EDTA) on ice for 30 minutes. After washing, the fluorescence intensity distributions were recorded using a laser scanning cytometer (LSC; see below).

❖ *Determination of background fluorescence*: Following the first LSC measurement, the agarose embedded Dynabead samples were equilibrated with DNase I buffer (10 mM Tris-HCl pH 8.0, 0.1 mM $CaCl_2$, 2.5 mM $MgCl_2$) and digested with DNase I at a final concentration of 5 μg/ml in 300 μl DNase I buffer at 37°C for 60 minutes. After enzymatic treatment, the agarose-embedded beads were washed with 500 μl ice cold 1×PBS/EDTA and measured again by LSC in a second run, performed on the same beads. After correction for the average fluorescence of empty beads, the mean fluorescence intensity remaining after DNase digestion was subtracted from the mean of the first LSC run, to correct for the background fluorescence.

❖ *Automated microscopy*: Automated microscopic imaging was performed using an iCys instrument (iCys® Research Imaging Cytometer; CompuCyte, Westwood, Massachusetts, USA). A488 and PI were excited using a 488 nm Argon ion laser. The fluorescence signals were collected via an UPlan FI 40× (NA 0.65) objective. A488 was detected through a 530/30 nm filters, PI was detected through a 650/LP nm filter. Each field was scanned with a step size of 0.5 μm. Data evaluation and hardware control were performed with the iCys 7.0 software for Windows XP.

Supplementary Tables

Sample	Labeling	Synchronization in G1 phase	Mean of labeled fibers (%)	SD of labeled fibers (%)	Statistics related to
gDNA	R-loop	-	47.4	11.7	Fig. S1C
	TdT	-	58.0	5.4	Fig. S1A
	Pol. I - N	-	47.7	8.4	Fig. 1C
	Pol. I - L	-	59.4	6.0	Fig. 1A
		+	36.1	2.2	Fig. 1B
chr XII	Pol. I - N	-	53.4	7.2	Fig. 3E
		+	29.9	4.9	Fig. 3G
	Pol. I - L	-	19.9	8.0	Fig. 3D
		+	25.5	5.8	Fig. 3F

Table S1: Frequency of labeled fibers among combed DNA molecules. Tables S1-S5 are related to Figures 1, 3 and Supplementary Figure S1. Labeled fibers were counted and divided by the total number of fibers in multiple vision fields. The table shows the means ± standard deviations of the appropriate percentages. R-loop: S9.6 labeling; TdT: Labeling of free 3'OHs in tailing reaction by terminal deoxynucleotidyl transferase; Pol. I – N and Pol. I – L: nick translation by *E. coli* DNA polymerase I under conditions limiting (L) or not limiting (N) incorporation of the labeled nucleotide into the vicinity of the nicks. Statistics are based on ~300 fibers for each sample. (For further details see Materials and Methods of main article.)

Labeling	Mean of labeled ends (%)	SD of labeled ends (%)	Statistics related to
R-loop	33.9	8.0	Fig. S1C
TdT	29.0	7.1	Fig. S1A
Pol. I - L	53.4	13.0	Fig. 1A

Table S2: Frequency of fiber termini with nick or R-loop labeling. The percentages of labeled gDNA ends in a vision field after DNA combing of the samples shown in Figure S1C (R-loop), Figure S1A (TdT) and Figure 1A (Pol I – L) are represented. Labeled ends were counted and divided by the total number of DNA ends in multiple vision fields. The table shows the means and standard deviations of the appropriate percentages. Statistics are based on ~300 fibers for each sample.

Combed DNA	gDNA		chr XII	
	**Non-synchronized**	**G1-synchronized**	**Non-synchronized**	**G1-synchronized**
Mean fragment length carrying one nick (kb)	72.4	97.8	99.9	154.6
Standard deviation of fragment length carrying one nick (kb)	19.8	25.9	21.8	18.2
Statistics related to	Fig. 1A	Fig. 1B	Fig. 3D	Fig. 3F

Table S3: Nick density measured in combed gDNA and chr XII DNA of non-synchronized and G1 synchronized cells. Nicks labeled by limited nick translation were counted and divided by the total length of DNA on multiple vision fields. Statistics are based on ~300 fibers for each sample.

Sample	Polymerase labeling	Number of fibers measured	Total length of fibers measured (µm)	Average fiber length (µm)	SEM	Statistics related to
gDNA	Limited	113	4066	36.0	3.5	Fig. 1A
gDNA - G1	Limited	118	3764	31.9	3.4	Fig. 1B
gDNA	Non-limited	114	4423	39.1	3.1	Fig. 1C
λ DNA	---	170	1877	11.0	4.4	Fig. 1F
λ DNA + Nickase	---	211	1175	5.6	3.3	Fig. 1G

Table S4: Fiber lengths statistics of nick-labeled gDNA and of λ DNA. Fiber lengths were measured and for the calculation of average fiber lengths the total lengths were divided by the total numbers of fibers in multiple vision fields. The table shows the means and SEM of the average lengths.

Sample	Polymerase labeling	% of fibers labeled at one end	% of fibers labeled on both ends	% of fibers labeled internally	Statistics related to
gDNA	Limited	37.8	21.7	8.3	Fig. 1A
gDNA - G1	Limited	27.2	8.7	6.8	Fig. 1B
gDNA	Non-limited	35.7	11.2	3.4	Fig. 1C
chr XII	Limited	19.9	0	2.1	Fig. 3D
chr XII	Non-limited	50.9	2.5	5.0	Fig. 3E

Table S5: Percentage of end-labeled and internally labeled fibers. Fibers labeled on one or both ends were counted and divided by the total number of fibers counted. Labeling of free 3’OHs was performed by *E. coli* DNA polymerase I under conditions limiting or not limiting incorporation into the vicinity of the nicks. All internally labeled fibers were also labeled on at least one end.

Supplementary Figures

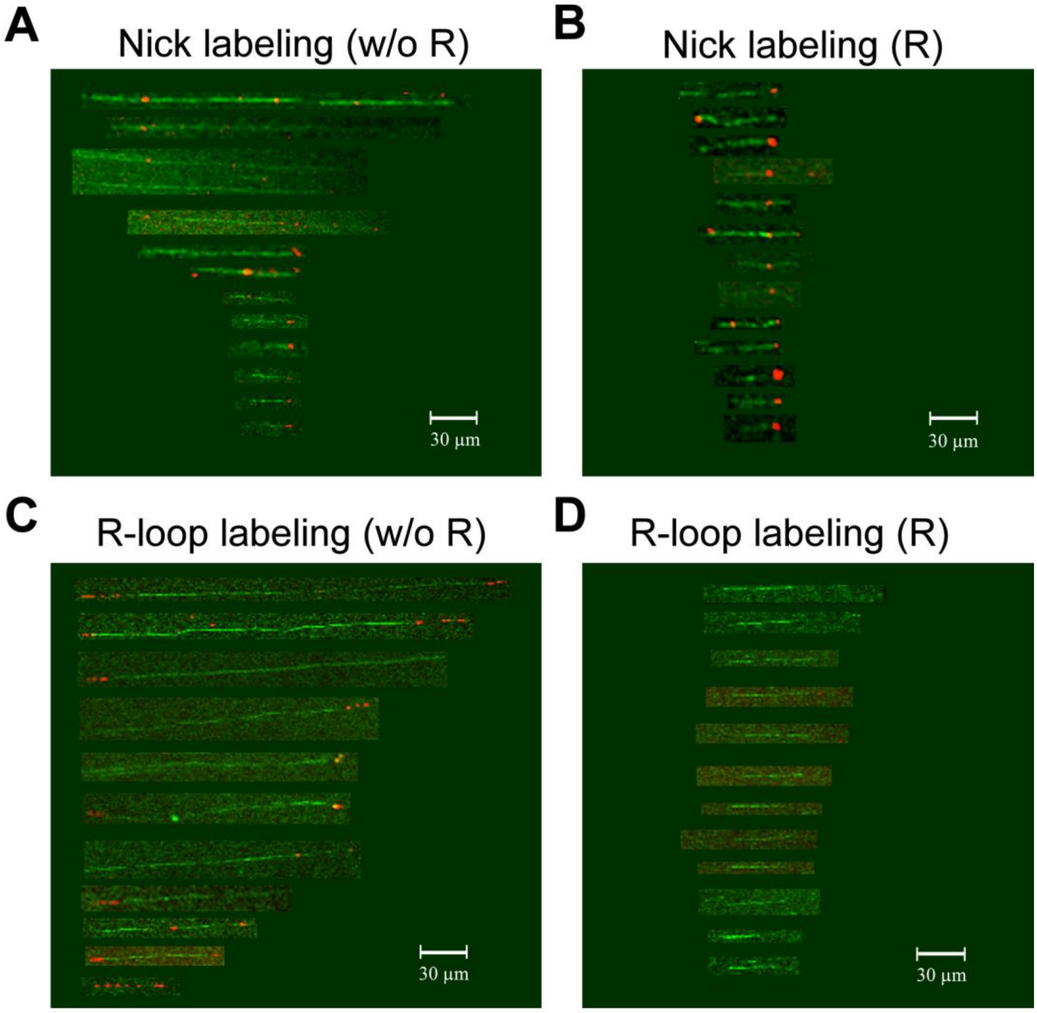

Figure S1 (related to Figure 1): Detection of R-loops and nicks on combed gDNA from non-synchronized *S. cerevisiae* cells (BY4741), without or with RNase treatment. Biotinylated nucleotides were incorporated by TdT into agarose-embedded gDNA. Plugs were further incubated either without (w/o R) or with a combination of RNases (R) to remove any tails that could be primed by free 3'OHs of RNA hybridized to DNA (see Materials and Methods in main text). Primary antibodies were anti-biotin (for nick labeling) or S9.6 (for R-loop labeling). The secondary antibody was AlexaFluor 647-conjugated anti-mouse IgG (red) in both cases. DNA molecules were stained with YOYO-1 (green). **(A)** nick-labeled combed gDNA without RNase digestions; **(B)** nick labeling of combed gDNA after digestion with the combination of RNAses (i.e. RNase A, then RNase HI, then human RNase H2; see Materials

and Methods in main text); **(C)** R-loop labeling of combed gDNA without RNase digestions; **(D)** R-loop labeling of combed gDNA after digestion with the combination of RNases. Fluorescent labeling of nicks and R-loops were carried out in separate gDNA samples that originated from the same cell sample.

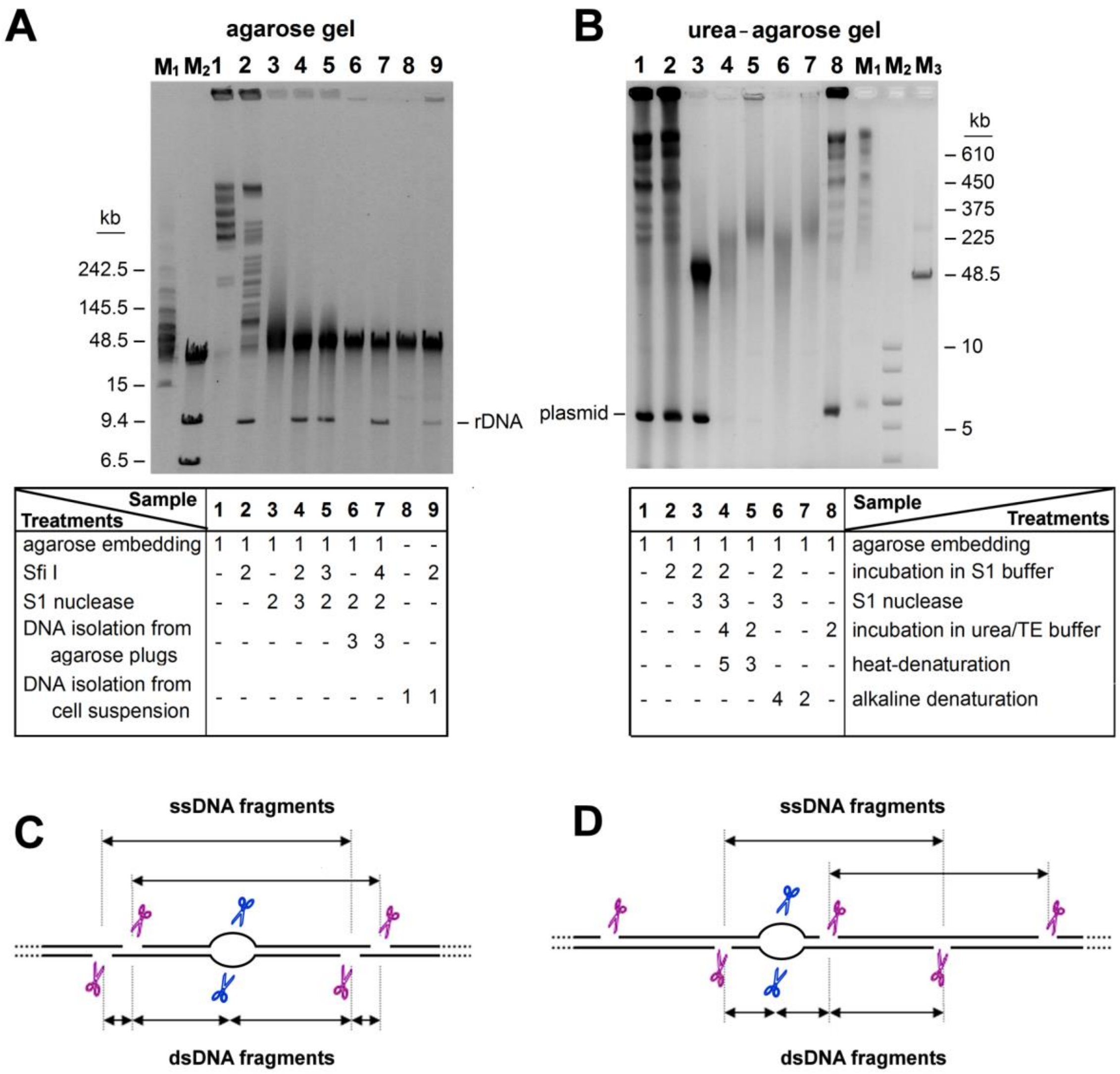

Figure S2: Gel electrophoretic analysis of *S. cerevisiae* gDNA demonstrates nicks arranged on the two DNA strands in a non-apposed manner. **(A)** Discontinuities and ss stretches were converted to ds breaks in agarose-embedded or isolated gDNA of WDHY199 *S. cerevisiae* cells using S1 nuclease and the cleavage products were analyzed on non-denaturing agarose gels. The gels were run in FIGE mode resolving also the higher molecular weight DNA fragments of gDNA. Lanes: M_1. Midrange marker (undenatured); M_2. Hind III-digested λ DNA (undenatured); 1. non-treated, agarose-embedded gDNA; 2. agarose-embedded gDNA cleaved with Sfi I; 3. gDNA digested with S1 nuclease in agarose plugs; 4. Sfi I cleavage followed by S1 nuclease digestion of gDNA in agarose plugs; 5. S1 nuclease digestion

followed by Sfi I cleavage of gDNA in agarose plugs. In lanes 6-7 the gDNA was digested with S1 nuclease in the plug, then either extracted from the plug (lane 6), or extracted from the plug then cleaved with Sfi I (lane 7). In lanes 8-9 the gDNA was extracted from ethanol fixed cells without embedding, without (lane 8), or with (lane 9) further cleavage with Sfi I. The table below the panel shows the order of treatments. **(B)** Urea/heat- or alkaline denaturation and analyses of the ss DNA on urea-agarose gels to detect ss breaks in WDHY199 *S. cerevisiae* gDNA by FIGE. Lanes: 1. undenatured gDNA; 2. undenatured gDNA after incubation of the agarose plugs in S1 buffer; 3. undenatured gDNA after S1 nuclease digestion; 4. urea/heat-denatured, S1 digested gDNA; 5. urea/heat-denatured gDNA; 6. alkaline-denatured, S1 nuclease-digested gDNA; 7. alkaline-denatured gDNA; 8. gDNA incubated in urea buffer; M_1. Pulse marker (225-2200 kb; undenatured); M_2. 1 kb marker (undenatured); M_3. λ DNA (undenatured). FIGE was carried out in urea-agarose gel resolving ss and ds fragments in the 5-1000 kb range. Plasmid: 2-micron plasmid. The table below the panel shows the order of treatments. **(C-D)** Possible arrangements of nicks along chromosomes. Scissors represent S1 nuclease used to transform nicks (pink) and other ss regions (blue) into ds fragments before denaturation. The double-headed arrows represent the categories of fragments anticipated according to the two scenarios considered. If nicks are present at loop-size intervals close to each other on the complementary strands, denaturation without S1 digestion and following S1 digestion would both yield approximately loop-size ss fragments. If nicks were present independently from each-other on the two strands, the ss fragment size would be ~twice larger without S1 digestion than after S1. The presence of S1 sensitive ss regions would blur these differences only if they were present at a comparable frequency and arranged independently from the nicks. The picture seen in panel B is best described by model D.

In the case of agarose-embedded samples the individual chromosomes were resolved without any sign of fragmentation or degradation (panel A, lane 1). Digestion of the agarose-embedded gDNA with the rare-cutter Sfi I with a single recognition site within the rDNA units allowed the simultaneous visualization of these 9.1 kb fragments; nonspecific DNA degradation was not revealed in this internal control (panel A, lane 2). When the deproteinized DNA of agarose-embedded cells were digested with S1 nuclease, ds fragments of ~50 kb average size were observed (panel A, lane 3). A sharp 9.1 kb rDNA band appears in the S1 plus Sfi I digested samples (panel A, lanes 4 and 5) independently of the order of

treatments, demonstrating the lack of any significant non-specific DNA degradation by S1. At the concentration of the S1 nuclease used, nickase generated nicks were completely converted into ds breaks in similar conditions (data not shown). When gDNA was isolated from suspensions of *S. cerevisiae* spheroplasts, the size of the DNA fragments obtained gave a peak around 50 kb (panel A, lanes 8-9), that is below the size of the smallest *S. cerevisiae* chromosome.

After heat treatment in the presence of 8 M urea (see Materials and Methods and [6]), the size of ss gDNA fragments was higher for the DNA sample which was denatured without prior S1 digestion relative to the ss fragments of the denatured, S1-predigested sample (compare lane 4 and 5 in panel B). Thus, endogenous nicks arranged in a disjoint manner on the complementary strands might serve as predilection points for S1 cleavage (see [7] and panels C-D for further explanation). The 2-micron plasmid [8], in denatured state, ran out of the gel (compare lanes 1-3 and 8 with 4-7 in panel B), since small ss fragments migrate faster in urea-agarose gels [9]. Urea or heat alone could not reveal or cause discontinuities (see [9] and panel B, lane 8). Alkaline denaturation gave similar results as urea/heat-denaturation (; panel B, compare lanes 6-7 with lanes 4-5). Thus, the fragmentation of the DNA in alkaline conditions cannot be attributed to the presence of ribonucleotides in gDNA [10]. Note also that the agarose plugs were not melted during alkaline denaturation, so no mechanical breaks could have occurred.

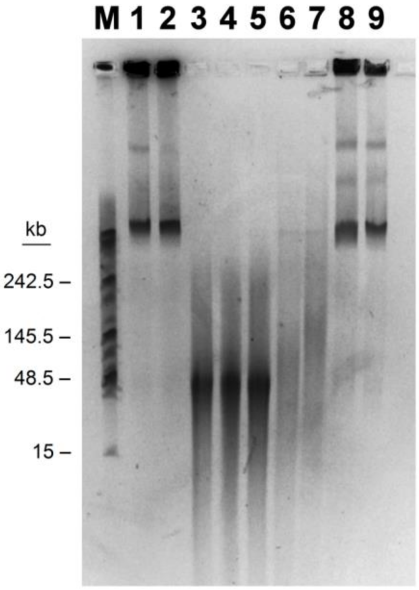

Treatments \ Sample	1	2	3	4	5	6	7	8	9
agarose embedding	1	1	1	1	1	1	1	1	1
incubation in S1 buffer	-	2	2	3	2	2	-	-	-
S1 nuclease	-	-	3	4	3	3	-	-	-
incubation in urea/TE buffer	-	-	-	2	4	4	2	-	2
heat-denaturation	-	-	-	-	-	5	3	2	-

Figure S3: S1 nuclease sensitive sites at loop-size intervals in the *S. pombe* genome. FIGE analysis of agarose-embedded gDNA of *S. pombe* cells (strain L972h^{-}). The different treatments of gDNA and the sequence of order within each treatment are summarized in the table underneath the gel. Lanes: M: Midrange marker (undenatured). 1. non-treated gDNA (control); 2. gDNA equilibrated in S1 buffer ; 3. S1 nuclease-digested gDNA; 4. gDNA pre-incubated in urea/TE buffer and then digested with S1 nuclease; 5. S1 digested gDNA equilibrated in urea/TE buffer; 6. urea/heat-denaturation of S1 digested gDNA; 7. urea/heat-denaturation of gDNA; 8. heat-denaturation of gDNA, without pre-incubation in urea/TE buffer; 9. gDNA incubated in urea/TE buffer.

The genome of *S. pombe* cells also harbor S1 nuclease sensitive sites at loop-size intervals. These also appear to be nicks arranged on the complementary strands in a non-apposed manner (see also arguments in conjunction with Figure S2).

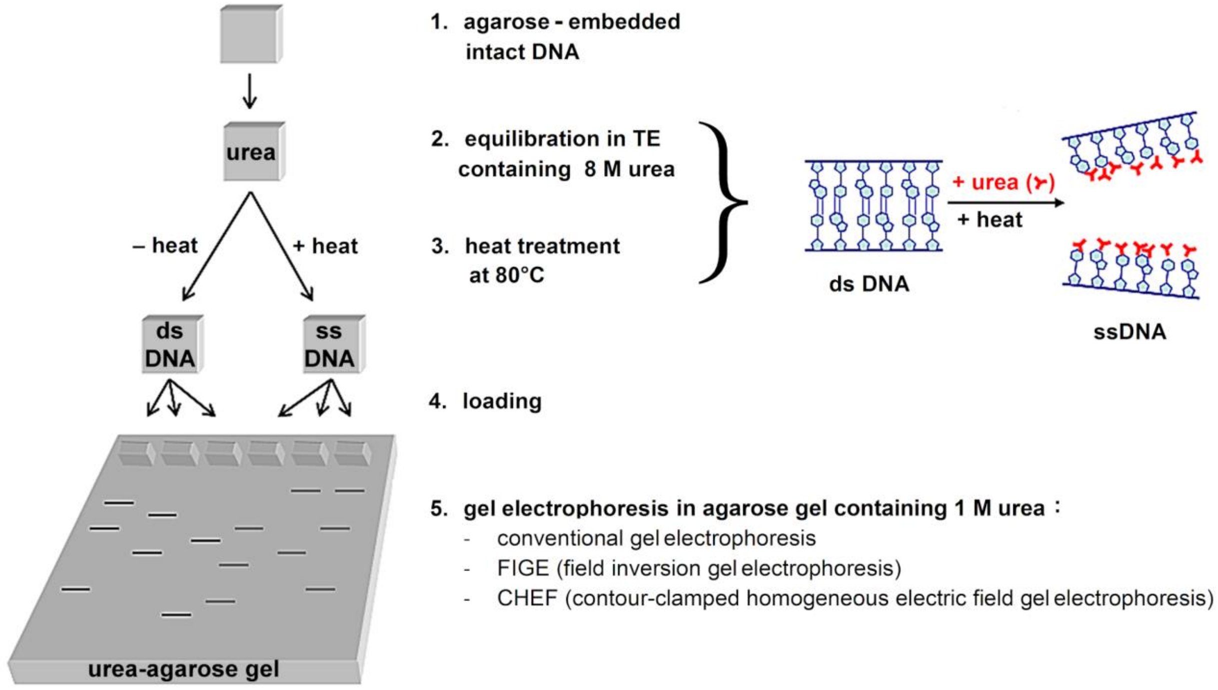

Figure S4: Work-flow of urea/heat-denaturing gel electrophoresis (related to Figure 5). For the detailed description of the experimental procedure see [9].

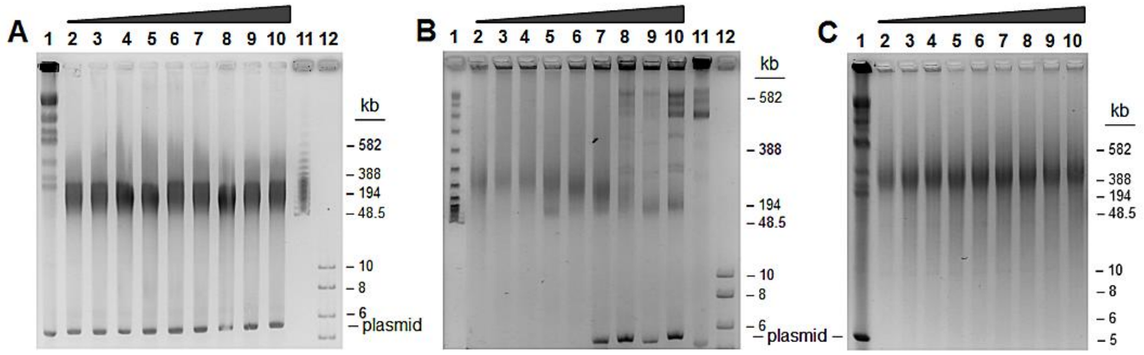

Figure S5: Effect of the superhelical state of gDNA on the incidence of ss discontinuities. Agarose plugs containing WDHY199 spheroplasts were treated with increasing concentrations of EBr and after lysis, the deproteinized, agarose-embedded DNA samples were digested with S1 nuclease (A), or urea/heat-treated at 80°C or 95°C in the presence of EBr (B and C, respectively). **(A)** FIGE analysis of gDNA of EBr-treated cells. Lanes: 1. gDNA from untreated spheroplasts (control); 2-10. gDNA of spheroplasts incubated in the presence of 0, 0.5, 1, 2.5, 5, 10, 25, 50, and 100 μg/ml EBr, respectively, then digested with S1 nuclease; 11. Pulse marker (50-1000 kb; undenatured); 12. 1 kb marker (undenatured). **(B)** Urea-agarose FIGE analysis of gDNA of EBr-treated spheroplasts. Lanes: 1. undenatured Pulse marker; 2-10. gDNA of spheroplasts incubated in the presence of 0, 0.5, 1, 2.5, 5, 10, 25, 50 and 100 μg/ml EBr, respectively, after urea/heat-denaturation at 80°C; 11. undenatured gDNA; 12. undenatured 1 kb marker. **(C)** Urea-agarose FIGE analysis of gDNA of EBr-treated spheroplasts. Lanes: 1. undenatured gDNA; 2-10. gDNA of cells incubated in the presence of 0, 0.5, 1, 2.5, 5, 10, 25, 50 and 100 μg/ml EBr, respectively, after urea/heat-denaturation at 95°C.

The dependence of Tm on fragment length was calculated based on the phenomenological model in [11]. Using that algorithm, the predicted melting point of a 25 bp random sequence would be ~68°C, while for a 15 bp random sequence it would be ~59°C. If such short intervals delimited the nicks on the complementary strands, and the melting temperature would be raised by about 10°C in the presence of EBr [12], denaturation at 80°C would still be allowed.

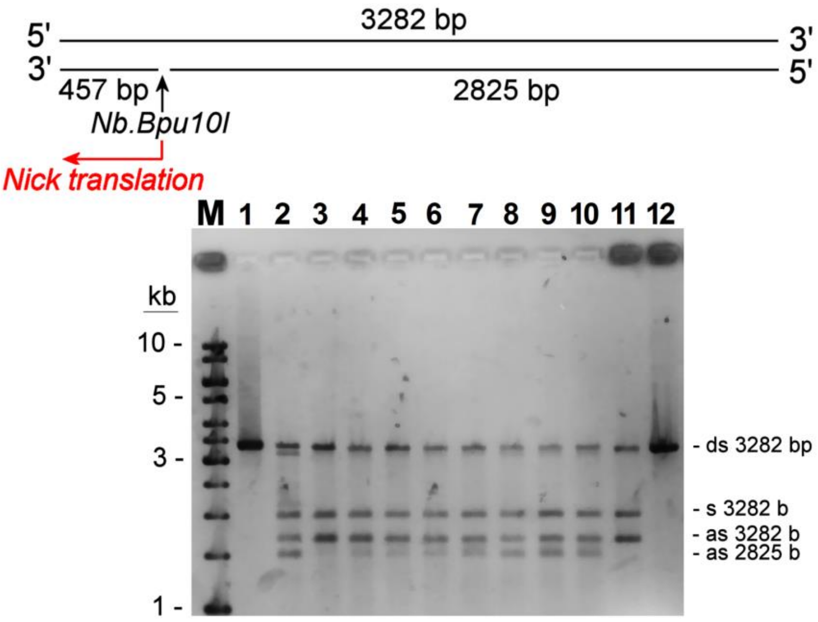

Treatments \ Sample	1	2	3	4	5	6	7	8	9	10	11	12
Nb.Bpu10I	+	+	+	+	+	+	+	+	+	+	-	-
agarose embedding	+	+	+	+	+	+	+	+	+	+	-	-
nick translation:	-	-	+	+	+	+	+	+	+	+	-	
dNTP (µM)	-	-	5	5	5	5	5	0.5	1	2.5	-	-
ddNTP (µM)	-	-	0	0.5	1	2.5	5	5	5	5	-	-
urea/heat-denaturation	-	+	+	+	+	+	+	+	+	+	+	-

Figure S6: Optimization of dNTP/ddNTP ratio for nick translation in limiting conditions. EBr-stained gel. Lane M: 1 kb marker (undenatured); lanes 1-2: Nb.Bpu10I digested, agarose-embedded 3282 bp long PCR product, before (1) and after (2) urea/heat-denaturation; lanes 3 -10: urea/heat-denatured, Nb.Bpu10I digested and agarose-embedded PCR products after nick translation using the nucleotide concentrations given in the table. Lanes 11-12: mixture of 500 ng urea/heat-denatured (11) and 500 ng non-denatured (12) PCR product without agarose-embedding. ds: double stranded PCR products; s: sense strand of the PCR products; as: antisense strand of the PCR products. Notes: The complementary strands of the PCR product run separately due to their different nucleotide composition [9]. The 457 bp fragments were allowed to run out of the gel. Both urea/heat-denaturation and Nb.Bpu10I digestion were partial so as to be able to visualize all the components of the reaction. The ratio of dNTP/ddNTP used in lane 9 was considered optimal and used throughout labeling in limited conditions.

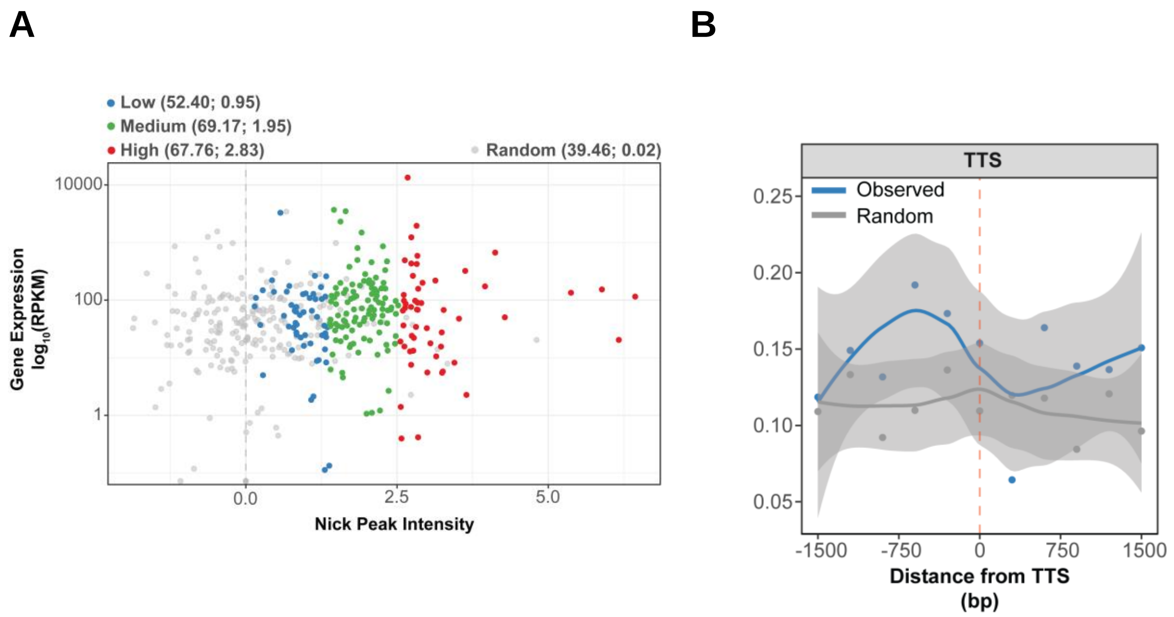

Figure S7 (related to Figures 2, S8 and S9): Nick incidence correlates with gene expression at TSSs but is not related to TTSs. **(A)** Scatterplot showing the correlation between nick peak intensity (X-axis) vs. gene expression (Y-axis) of the closest gene. Nick ChIP-chip binding sites were grouped into three categories based on intensity (i.e. ChIP enrichment): low (blue), medium (green) and high (red). The median RPKM of the three categories are in parenthesis. Gray points represent random genomic regions. **(B)** Nicks are not enriched at transcription termination sites (TTSs; n (total gene pool) = 6664). The average binding intensity of nick ChIP-chip signals is shown as a scatterplot for observed (blue) and randomly permutated (gray) nick peaks (+/- 1500 base pairs). Lines represent the smoothed mean after loess normalization. Faded area shows the 95% confidence interval for predictions from the loess model. Significant difference could not be assigned using two-sided Student t-test.

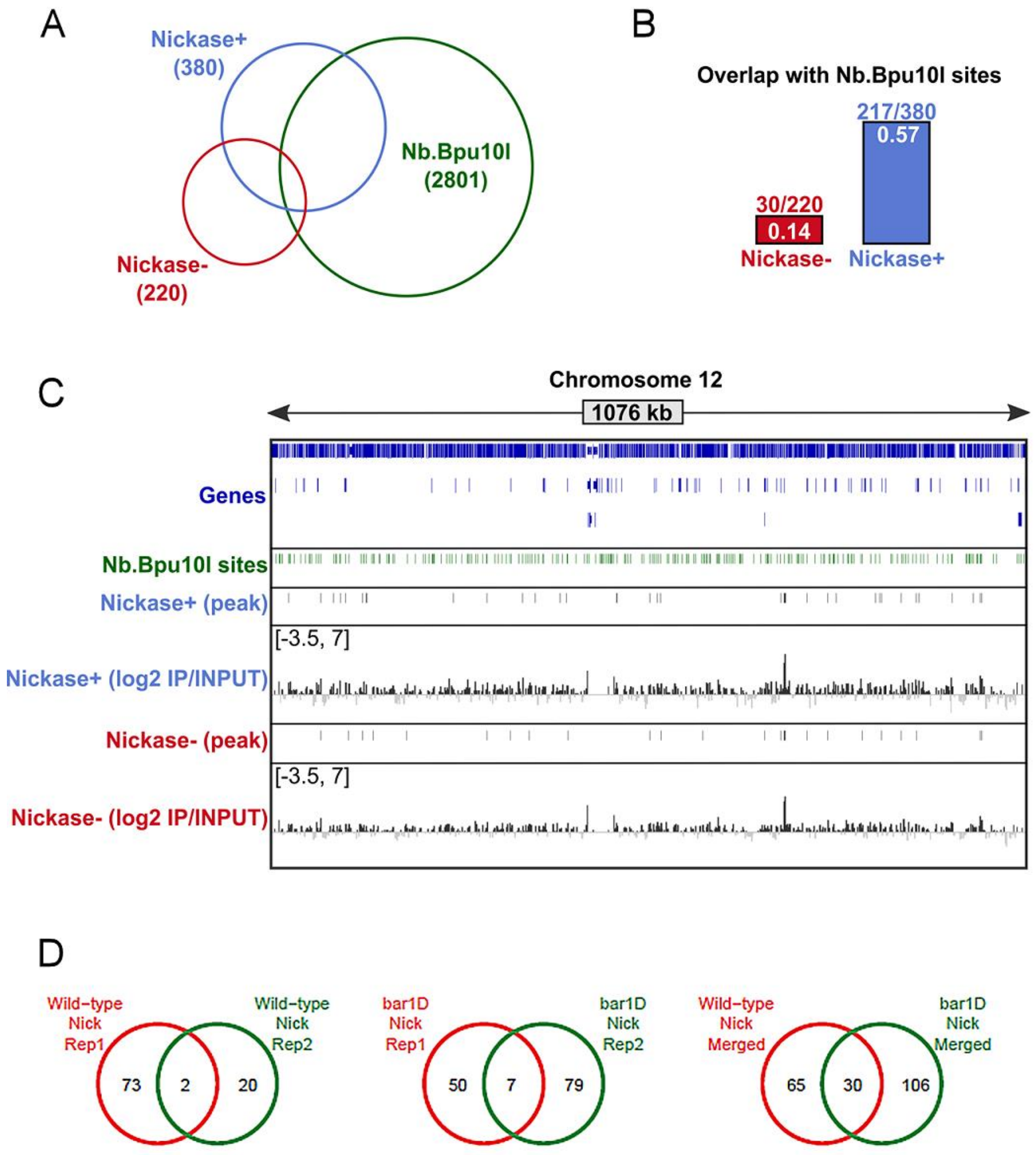

Figure S8 (related to Figures 2, S7 and S9): Nick ChIP-chip analyses of endogenous nicks and exogenous nicks. **(A)** Proportional Venn diagram showing the overlapping nick sites with predicted Nb.Bpu10I (nickase) recognition sequences (green) in control (Nickase-; red) and

Nb.Bpu10I nickase-treated (Nickase +; blue) chip samples. **(B)** The proportion of nicks that overlaps with Nb.Bpu10I cleavage sites is significantly higher in the nickase + than nickase - sample (two-proportion z test, $p<0.0001$). **(C)** Representative genome browser tracks showing the distribution of nicks and Nb.Bpu10I cleavage sites along chr XII. Tracks from top to bottom, Genes: protein coding ORFs along chromosome XII, NbBpu10I sites: position of Nb.Bpu10I cleavage sites predicted from the distribution of 5'-CCTNAGC-3' recognition motifs, Nickase + (peak): nicks identified in nickase-treated samples, Nickase + (log2 IP/INPUT): ChIP signal of the nickase-treated sample. Nickase– (peak): position of endogeneous nicks identified in control (no nickase) samples. Nickase– (log2 IP/ INPUT): ChIP signal of the control (no nickase) sample. Minimum and maximum ChIP signal intensities are indicated within brackets. **(D)** Comparison of two replicates of two independent biological samples (REP1 and REP2) of Nick ChIP-chip experiments carried out in *S. cerevisiae* strains wild-type (BY4741) and the isogenic *bar1Δ* mutant, which was G1-synchronized (see Materials and Methods). The counts represent the number of nick peaks in the upper quartiles of nick signal intensities.

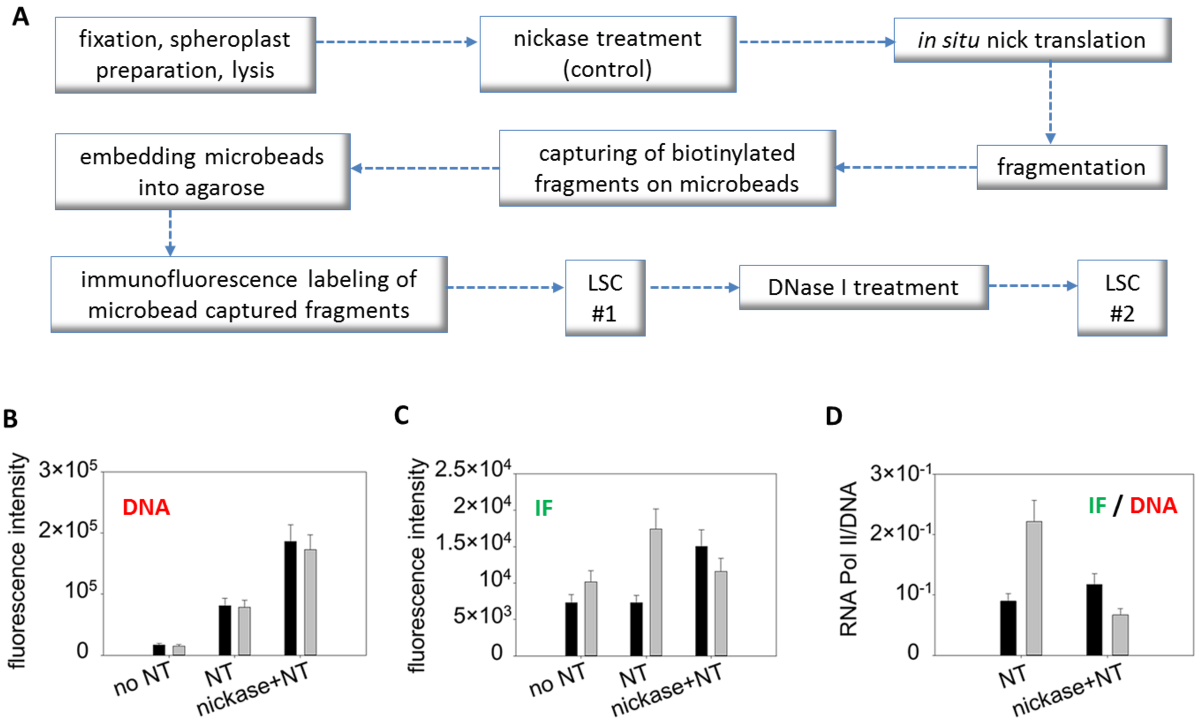

Figure S9 (related to Figures 2, S7 and S8): Global chip-on-beads analyses of nick – RNAP II colocalization. **(A)** Flow-chart of the experiment. LSC: laser scanning cytometry. **(B)** Mean EBr fluorescence intensity (DNA) of microbead-captured chromatin fragments without nick translation (no NT), and nick-translated chromatin fragments without (NT) or with nickase treatment (nickase+NT). Fragments were labeled either with RNAP II Ser2-P- (black columns), or RNAP II Ser5-P- (grey columns) specific antibodies. The differences between the first and the second LSC measurements (LSC#1-LSC#2; see Supplementary Materials and Methods) were plotted on the Y-axis. **(C)** Mean immunofluorescence (IF) intensities of the same samples as in panel B. **(D)** Mean immunofluorescence intensities of panel C normalized to the mean DNA amount captured by the microbeads shown in panel B (IF/DNA). Error bars represent SD of ~2000 beads measured by LSC. One representative of two independent measurements is shown in the Figure. The DNA-normalized RNAP II immunofluorescence is similar in the samples with and without nickase treatment in the case of Ser2-P, while the Ser5-P signals are much higher when the endogenous nicks were labeled only.

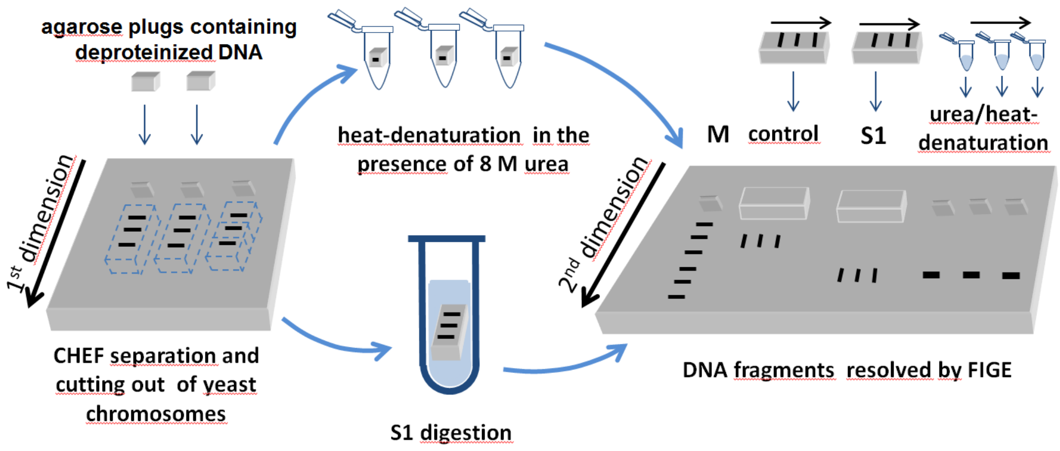

Figure S10 (related to Figures 3 and S11): Scheme of the two-dimensional gel electrophoretic analyses. For a detailed description of the experimental procedure see Supplementary Materials and Methods.

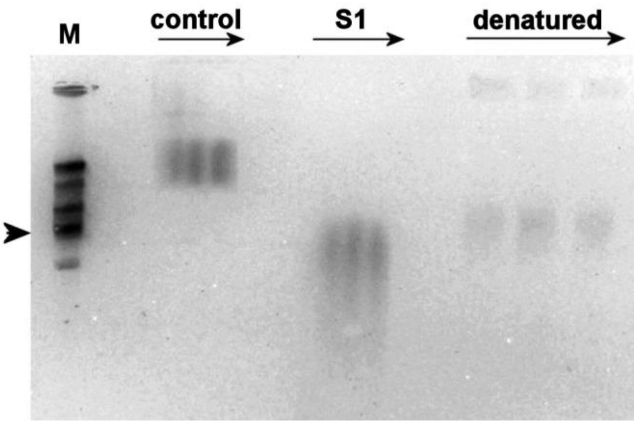

Figure S11 (related to Figures 3 and S10): Two-dimensional gel electrophoretic analyses of nicks in *S. pombe* (strain: L972 h-) chromosomes. Chromosomal DNA molecules (3.5 - 5.7 Mb) were first resolved by CHEF (not shown in the image), then the bands containing the individual chromosomes were cut out and either treated with S1 nuclease (labeled 'S1') or were urea/heat-denatured without S1 digestion (labeled: 'denatured'). Electrophoresis in the 2nd dimension (shown in the image) by FIGE was conducted in denaturing conditions. M: Midrange marker, undenatured. The arrow head points at the 48 kb DNA band. The horizontal thin arrows point from the large to the small chromosomes (chr I, II and III, from left to right).

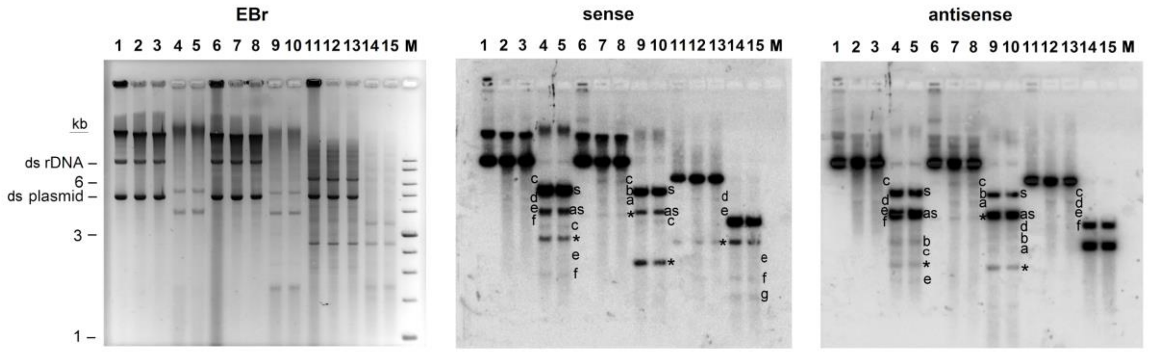

Treatments \ Sample	1	2	3	4	5	6	7	8	9	10	11	12	13	14	15
RNase H1	-	-	+	-	+	-	-	+	-	+	-	-	+	-	+
S1 nuclease	-	+	+	-	-	-	+	+	-	-	-	+	+	-	-
Sma I	+	+	+	+	+	-	-	-	-	-	-	-	-	-	-
Mlu I	-	-	-	-	-	+	+	+	+	+	-	-	-	-	-
Hind III	-	-	-	-	-	-	-	-	-	-	+	+	+	+	+
urea/heat-denaturation	-	-	-	+	+	-	-	-	+	+	-	-	-	+	+

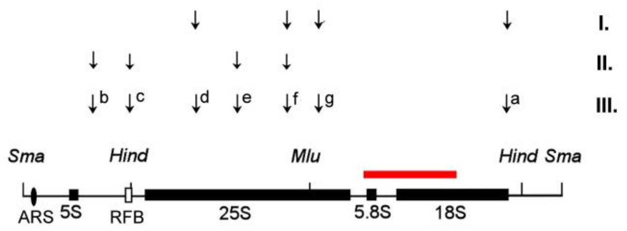

Figure S12 (related to Figures 4, S13 and S14): Mapping of DNA breaks within the rDNA locus using single-stranded rDNA probes. Southern blot analyses of agarose-embedded gDNA derived from WDHY199 *S. cerevisiae* spheroplasts. EBr stained gel (left panel), sense probe-hybridized (middle panel), and antisense probe-hybridized blots (right) of agarose-embedded samples containing gDNA after restriction endonuclease digestion and S1 nuclease treatment and/or urea/heat-denaturation (as summarized in the table). Lanes: 1. Sma I cleavage; 2. S1 nuclease digestion then Sma I cleavage; 3. RNase H1, then S1 nuclease digestion, then Sma I cleavage; 4. urea/heat-denaturation after Sma I cleavage; 5. RNase H1 and Sma I digestion, then urea/heat-denaturation. 6. Mlu I cleavage; 7. S1 nuclease digestion then Mlu I digestion; 8. RNase H1, then S1 nucleaase digestion, then Mlu I cleavage; 9. urea/heat-denaturation after Mlu I digestion; 10. urea/heat-denaturation after RNase H1 and Mlu I digestion. 11. Hind III cleavage; 12. S1 nuclease digestion, then Hind III cleavage; 13. RNase H1, then S1 nuclease digestion, the Hind III cleavage; 14. urea/heat-denaturation after Hind III digestion; 15. urea/heat-denaturation after RNase H1 and Hind III digestion. M: 1 kb marker (undenatured); ds plasmid: non-denatured 2-micron plasmid; ds rDNA: non-denatured 9.1 kb rDNA units. s and as: separately migrating 9.1 kb sense and antisense strands of denatured rDNA units. a-g: fragments derived from endogenous nicks after S1 nuclease digestion or urea/heat-treatment; *: artefactual fragments generated by the nicking activity of the given restriction endonuclease. Scheme: Location of nicks in rDNA. I.: nicks detected

with the sense strand-specific probe in denatured, single-stranded rDNA samples; II.: nicks detected with the antisense strand-specific probe in denatured, single-stranded rDNA samples; III.: nicks converted to double strand breaks with S1 nuclease in non-denatured, double-stranded rDNA samples, detected with either sense or antisense strand specific probes. The scheme was constructed as in Figure 4 to indicate the mapped positions of nicks (a-g) in rDNA.

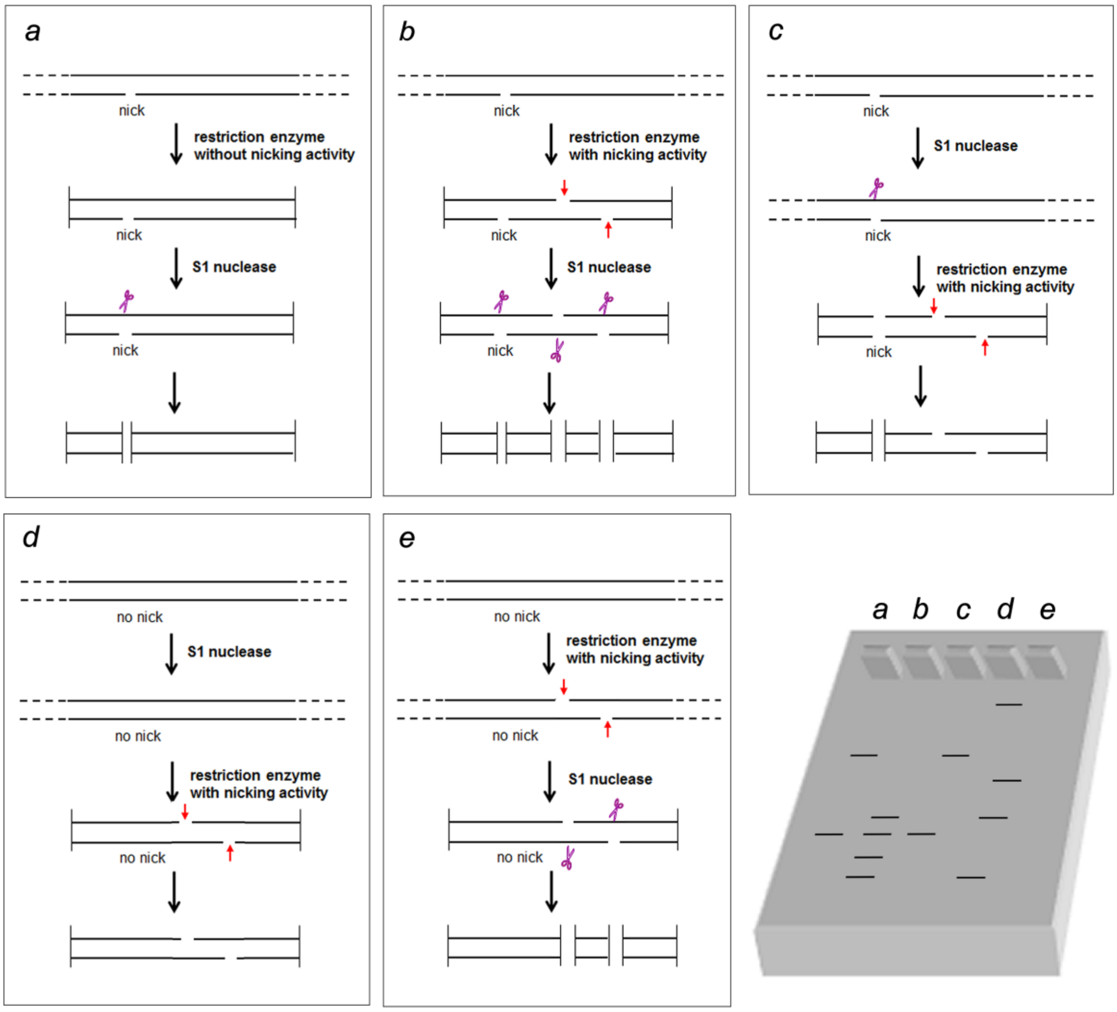

Figure S13: Dependence of the apparent fragment size on the order of treatments during detection of nicks in the rDNA units (related to Figures 4, S12 and S14). Endogenous nicks can be detected when digestion with the restriction enzyme without off-target nicking activity (e.g. Sfi I) is followed by S1 nuclease treatment (**a**), or when S1 nuclease treatment is followed by digestion with the restriction enzyme either with or without off-target nicking activity (**c**). Importantly, artefactual nicks generated by restriction enzymes with off-target activity can be transformed into DSBs upon S1 nuclease treatment (**b** and **e**). In the absence of endogenous nicks, the rDNA remains intact when S1 nuclease treatment is followed by the restriction enzyme either with or without off-target nicking activity (**d**). Red arrow: Off-target nicking by restriction enzyme. Scissors: S1 nuclease. The gel image shows the approximate migration of DNA bands expected after gel electrophoresis in each possible scenario (a-e).

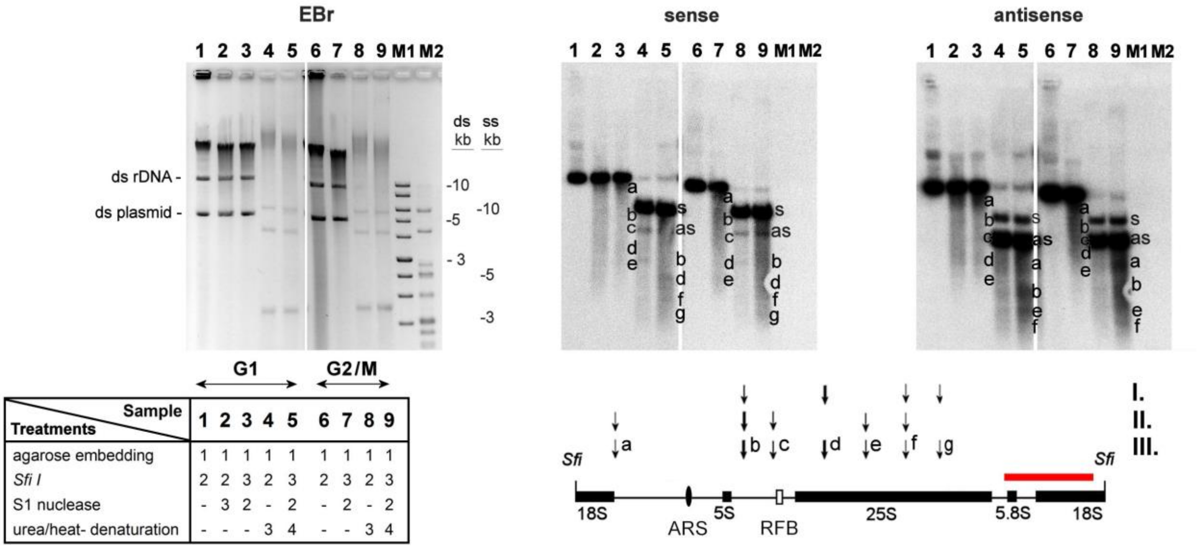

Sample / Treatments	1	2	3	4	5	6	7	8	9
agarose embedding	1	1	1	1	1	1	1	1	1
Sfi I	2	2	3	2	3	2	3	2	3
S1 nuclease	-	3	2	-	2	-	2	-	2
urea/heat- denaturation	-	-	-	3	4	-	-	3	4

Figure S14 (related to Figures 4, S12 and S13): Mapping of DNA breaks within the rDNA locus in yeast cells synchronized in G1 or G2/M phases of the cell cycle. The rDNA of deproteinized G1-synchronized *bar1Δ* and G2/M-synchronized BY4741 spheroplasts was analyzed by Southern blotting using strand specific rDNA probes. Left panel: EBr stained gel containing gDNA. The different treatments of gDNA and the sequence of order within each treatment are summarized in the table underneath the gel. Lanes: 1. and 6. Sfi I cleavage of gDNA from G1- and G2/M- cells, respectively; 2. Sfi I cleavage, then S1 nuclease digestion of gDNA from G1 cells; 3. and 7. S1 nuclease digestion, then Sfi I cleavage of gDNA from G1 and G2/M cells, respectively; 4. and 8. Sfi I cleavage, then urea/heat-denaturation of gDNA from G1 and G2/M cells, respectively; 5. and 9. S1 nuclease digestion, then Sfi I cleavage, then urea/heat-denaturation of gDNA from G1 and G2/M cells, respectively; M1 and M2. undenatured and denatured 1 kb marker, respectively. Plasmid: 2-micron plasmid; ds rDNA: non-denatured rDNA units; s and as: separately migrating sense and antisense strands, respectively, of the denatured rDNA units. Middle and right panels: Southern hybridizations using sense and antisense strand specific probes, respectively. a-g: fragments derived from endogenous nicks after S1 nuclease digestion or urea/heat-treatment. Scheme: Location of nicks in rDNA. I.: nicks detected with the sense strand-specific probe in denatured, single-stranded rDNA samples; II.: nicks detected with the antisense strand-specific probe in denatured, single-stranded rDNA samples; III.: nicks converted to double strand breaks with S1 nuclease detected with either sense or antisense strand specific probes in non-denatured,

double-stranded rDNA samples. The scheme was constructed as in Figure 4 to indicate the mapped positions of nicks (a-g) in rDNA.

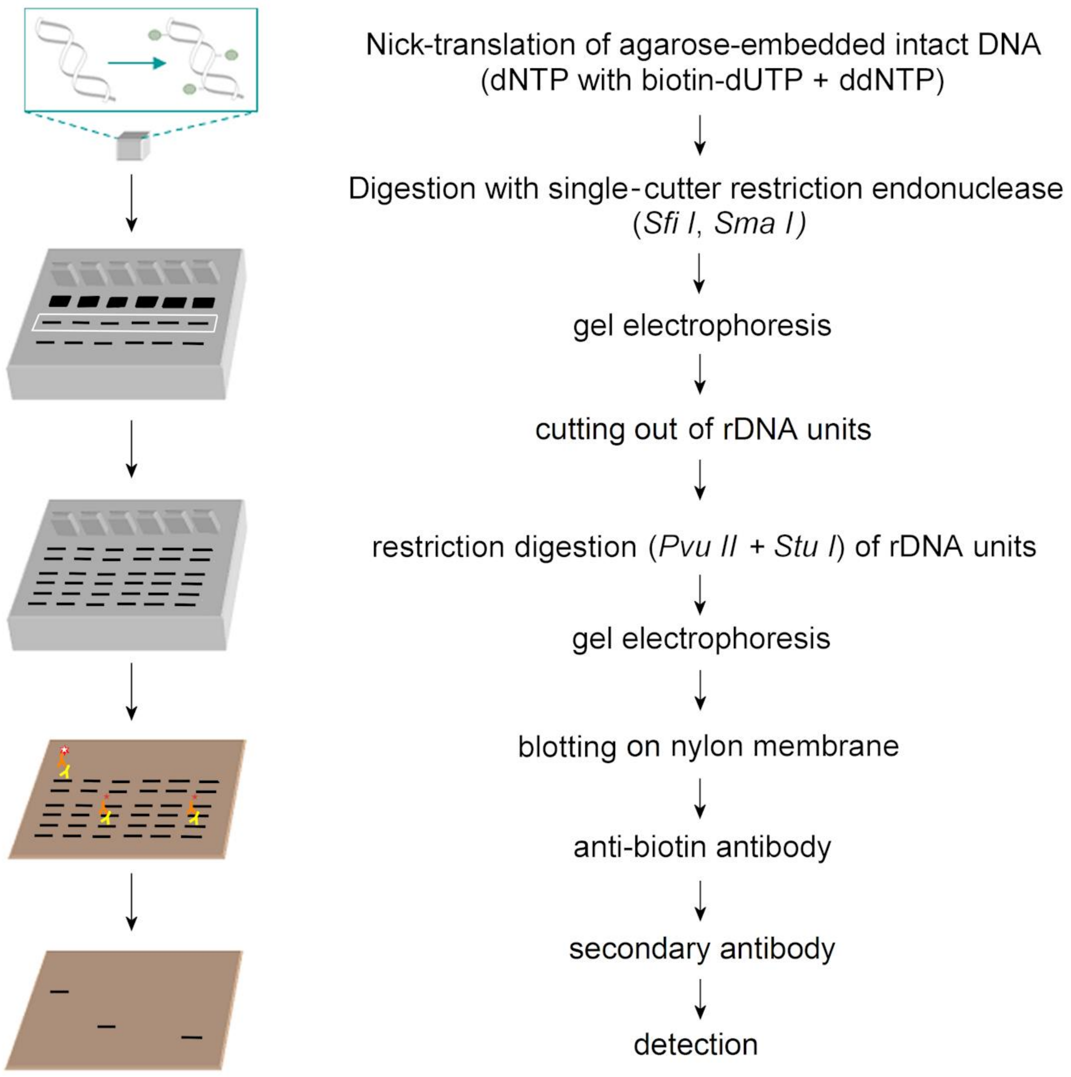

Figure S15 (related to Figures 5 and S16): rSW blot procedure for detection of nicks in rDNA units. For description of experimental procedure see Materials and Methods. Although the spatial resolution of the rSW method is limited by the restriction enzyme recognition sites present in the target sequence, the signal intensities give a quantitative assessment of the antibody recognized molecular entities. Moreover, the method provides an independent perspective on the incidence and location of nicks. Importantly also, it is devoid of the potential bias that could be associated with the amplification process preceding ChIP-seq analyses. A further advantage of the rSW method is that different features of the samples can be investigated on the same membrane and compared as each other's internal control (e.g. see Figure 5B for mapping of both nicks and R-loops on the same blot).

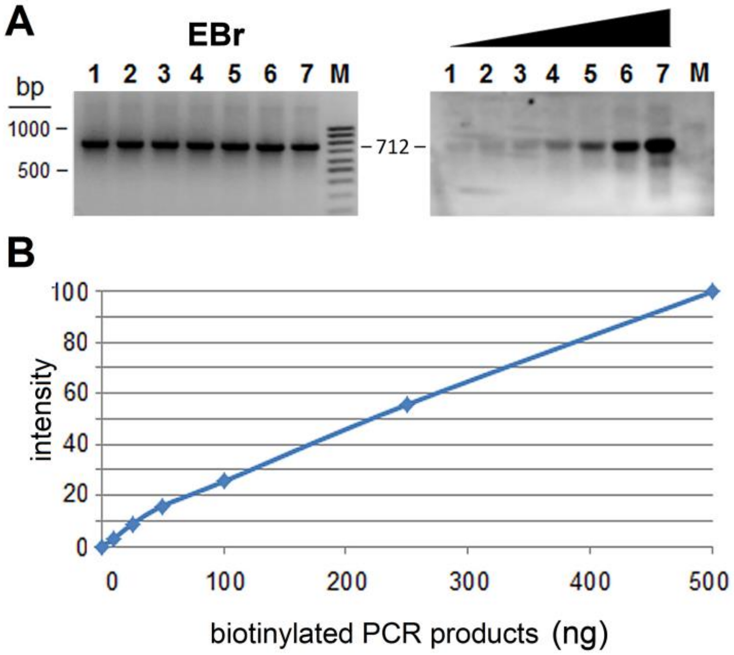

Figure S16 (related to Figures 5 and S15): Calibration of the rSW procedure. Identical amounts (500 ng) of a 764 bp PCR product containing biotinylated molecules at different ratios were analyzed on an 1 % agarose gel. The amount of biotinylated DNA molecules in the samples were 0 ng (lane 1), 10 ng (lane 2), 25 ng (lane 3), 50 ng (lane 4), 100 ng (lane 5), 250 ng (lane 6) and 500 ng (lane 7). Marker: 1 kb ladder (M). **(A)** Left sub-panel: EBr stained gel. Right sub-panel: rSW signal detected with anti-biotin antibody. **(B)**. densitometric evaluation. X-axis represents the amounts of biotinylated PCR products in ng. Y-axis represents rSW (biotin/EBr) signal intensity.

Supplementary References

1. Szekvolgyi, L., et al., *Nick-forming sequences may be involved in the organization of eukaryotic chromatin into approximately 50 kbp loops.* Histochem Cell Biol, 2006. **125**(1-2): p. 63-73.
2. Goyon, C. and M. Lichten, *Timing of molecular events in meiosis in Saccharomyces cerevisiae: stable heteroduplex DNA is formed late in meiotic prophase.* Mol Cell Biol, 1993. **13**(1): p. 373-82.
3. Lewinska, A., B. Miedziak, and M. Wnuk, *Assessment of yeast chromosome XII instability: single chromosome comet assay.* Fungal Genet Biol, 2014. **63**: p. 9-16.
4. Brugere, J.F., et al., *In-gel DNA radiolabelling and two-dimensional pulsed field gel electrophoresis procedures suitable for fingerprinting and mapping small eukaryotic genomes.* Nucleic Acids Res, 2000. **28**(10): p. E48.
5. Imre, L., et al., *Nucleosome stability measured in situ by automated quantitative imaging.* Sci Rep, 2017. **7**(1): p. 12734.
6. Materna, T., et al., *Electrophoretic separation of both single- and double-stranded nucleic acids in the same urea-containing agarose gel.* Anal Biochem, 1998. **255**(1): p. 161-3.
7. Hegedus, E., et al., *Heteroduplex analysis using flow cytometric microbead assays to detect deletions, insertions, and single-strand lesions.* Cytometry A, 2008. **73**(3): p. 238-45.
8. Chan, K.M., et al., *The 2 micron plasmid of Saccharomyces cerevisiae: a miniaturized selfish genome with optimized functional competence.* Plasmid, 2013. **70**(1): p. 2-17.
9. Hegedus, E., et al., *Separation of 1-23-kb complementary DNA strands by urea-agarose gel electrophoresis.* Nucleic Acids Res, 2009. **37**(17): p. e112.
10. Clausen, A.R., J.S. Williams, and T.A. Kunkel, *Measuring ribonucleotide incorporation into DNA in vitro and in vivo.* Methods Mol Biol, 2015. **1300**: p. 123-39.
11. Khandelwal, G. and J. Bhyravabhotla, *A phenomenological model for predicting melting temperatures of DNA sequences.* PLoS One, 2010. **5**(8): p. e12433.
12. Vardevanyan, P.O., et al., *Study of ethidium bromide interaction peculiarities with DNA.* Exp Mol Med, 2001. **33**(4): p. 205-8.
